# Implementing value-based healthcare: a scoping review of key elements, outcomes, and challenges for sustainable healthcare systems

**DOI:** 10.3389/fpubh.2025.1514098

**Published:** 2025-04-09

**Authors:** Hanan Khalil, Mary Ameen, Charles Davies, Chaojie Liu

**Affiliations:** ^1^Department of Public Health, La Trobe University, Bundoora, VIC, Australia; ^2^Faculty of Medicine, Nursing and Health Sciences, Clayton, VIC, Australia

**Keywords:** value-based health care, funding model, health care delivery innovations, patient centered approach, scoping review

## Abstract

**Introduction:**

Value-Based Health Care (VBHC) is an increasingly important healthcare paradigm that focuses on maximizing health outcomes relative to the cost of care delivered. Various healthcare organizations have adopted VBHC principles, but significant barriers remain in adapting care models, engaging stakeholders, and measuring outcomes. Moreover, the lack of standardized methods for measuring outcomes and financial sustainability further complicates the transition to VBHC. Understanding the factors that facilitate or hinder VBHC adoption is crucial to informing policy and practice for broader implementation. The objective is to map the literature addressing VBHC concerning population, study characteristics, funding models, outcome measures, and barriers and facilitators.

**Methods:**

Following the JBI methodology and the PRISMA-ScR reporting guidance, a scoping review was undertaken to include primary and secondary research on VBHC across various healthcare settings. Searches were undertaken in nine relevant databases. Peer-reviewed quantitative and qualitative studies published in English were included and analyzed. A total of 145 studies were included after screening 2,725 studies.

**Results:**

The findings show that the United States leads VBHC research, contributing 65% of the studies, followed by European countries. Cohort and cross-sectional studies were predominant, focusing on various populations, including hospitals, surgical patients, and cancer patients. Key findings highlight that Value-Based Purchasing and Time-Driven Activity-Based Costing models were the most frequently reported funding models. Traditional in-hospital care remains the dominant delivery model, with increasing interest in telemedicine. Outcome measure were diverse, ranging from patient-reported outcomes to cost savings for both patients and providers. Barriers to VBHC implementation include insufficient funding, fee-for-service model persistence, and resistance from healthcare professionals. Facilitators included strong leadership, multidisciplinary collaboration, and the use of digital tools.

**Conclusion:**

The review highlights the need for consistent outcome measurements, financial incentives, and improved data transparency to ensure the successful and scalable implementation of VBHC across healthcare systems. While VBHC shows promise in improving healthcare efficiency and quality, challenges remain in aligning financial and operational structures to fully support this paradigm shift.

## Introduction

Value-Based Health Care (VBHC) is an increasingly important healthcare paradigm that focuses on maximizing health outcomes relative to the cost of care delivered (1–4). In contrast to traditional fee-for-service models, which emphasize the quantity of services provided, VBHC aims to deliver higher quality, patient-centered care by aligning provider incentives with the value of outcomes achieved (5, 6). The rationale behind VBHC is rooted in improving both the efficiency and effectiveness of healthcare systems, fostering a shift from volume to value (7, 8). As healthcare costs rise globally and disparities in care delivery and care quality persist, implementing VBHC has become a priority for policymakers, providers, and payers alike (2, 9).

Despite the growing interest in VBHC, its implementation across different healthcare systems presents a complex challenge (2, 10–12). Various healthcare organizations have adopted VBHC principles, but significant barriers remain in adapting care models, engaging stakeholders, and measuring outcomes (1, 13). Previous studies have demonstrated that VBHC can lead to cost reductions and improved clinical outcomes; however, these benefits are not uniformly achieved across all settings (2, 14). The lack of standardized methods for measuring outcomes and financial sustainability further complicates the transition to VBHC. Understanding the factors that facilitate or hinder VBHC adoption is crucial to informing policy and practice for broader implementation.

This study aims to assess the barriers and facilitators of VBHC implementation across different healthcare settings by examining the findings from a wide range of studies. While many studies have reported on the potential of VBHC to improve quality and reduce costs, there is a gap in understanding the systemic and operational challenges that healthcare organizations face when implementing VBHC (2, 3, 15, 16). Identifying the key facilitators related to leadership, organizational structure, data infrastructure, care delivery processes, and patient-centered outcome measured—as well as barriers, such as resistance to change, funding constraints, processes, and data collection difficulties, will provide valuable insights for future initiatives. Given the complexity of healthcare systems and the variation in VBHC implementation across different countries and contexts, this study seeks to contribute to the evidence base by highlighting the real-world experiences of healthcare providers in their efforts to achieve value-based care outcomes by identifying the various funding models, delivery models, outcome measured and barriers and facilitators in implementing value-based healthcare. Therefore, the objective of this review is to map the literature addressing VBHC specifically in relation to the population involved, study characteristics, funding models, outcome measured and barriers and factors for implementing it. Therefore, the objective of this review is to map the literature addressing VBHC specifically in relation to the population involved, study characteristics, funding models, outcome measured, and barriers and factors for implementing it. Therefore, the objective of this review is to map the literature addressing VBHC specifically in relation to the population involved, study characteristics, funding models, outcome measures, and barriers and factors for implementing it. Therefore, the objective of this review is to map the literature addressing VBHC specifically in relation to the population involved, study characteristics, funding models, outcome measured, and barriers and factors for implementing it.

## Methods

This scoping review follows the JBI methodology outlined by Peters et al. (17) and Tricco et al. (18) and is registered in the Open Science Framework (17, 18). The review considers primary and secondary research on value-based healthcare (VBHC). Participants include anyone receiving or administering VBHC, with the concept focusing on any aspect of VBHC. The context is any healthcare setting, and the review includes peer-reviewed quantitative and qualitative studies published in English. Non-peer-reviewed studies are excluded. Inclusion criteria included studies published in English, focused on VBHC models in any healthcare setting, and providing detailed outcomes or implementation frameworks. Exclusion criteria included non-peer-reviewed studies and those lacking relevant VBHC-related data.

Search Strategy: An example search string used in Ovid MEDLINE included terms like ‘value-based care’ OR ‘value-based health care’ AND ‘outcome measures’ AND ‘healthcare systems’. Please refer to Appendix 1 for a complete list of searches.

The review employs a three-step search strategy. First, a limited search of Ovid MEDLINE identifies relevant text words and index terms. Second, a comprehensive search using all identified keywords and index terms is conducted across databases, including JBI EBP Database, EBM Reviews – Cochrane Database of Systematic Reviews, Embase, Global Health, Ovid MEDLINE (R), APA PsycInfo <1806 to November Week 2 2023>, Social Work Abstracts, EBM Reviews – Database of Abstracts of Reviews of Effects, EBM Reviews – Health Technology Assessment, and EBM Reviews – NHS Economic Evaluation Database. These searches were completed by 13 November 2023. Third, the reference lists of all identified reports and articles are examined for additional studies. The key search terms used include “value-based care,” “value-based health care,” “value-based model,” and “value-based framework.” A list of the searches undertaken is attached in Appendix 1. The searches covered studies published from 2000 to November 2023.

### Data screening, extraction and presentation

Data screening was done by four researchers (HK, CD, MA, and RA) for both titles and abstracts and full text in pairs. Any discrepancies were discussed. Relevant data are extracted from the included studies to address the review question, following Peters et al. (17). The data extracted include the author, country, participants involved, study type, VBHC models, funding models, delivery models, outcome measures, and barriers or facilitators. The extracted data are presented in a logical and descriptive summary that aligns with the review’s objectives. Sources were screened using a two-level process: title and abstract screening followed by full-text review. Discrepancies were resolved through discussion among the research team. Data extraction was conducted using a standardized form to capture author, country, VBHC model, funding, delivery models, and outcomes. Data synthesis was conducted using a narrative synthesis approach to summarize findings.

## Results

**Figure 1:**
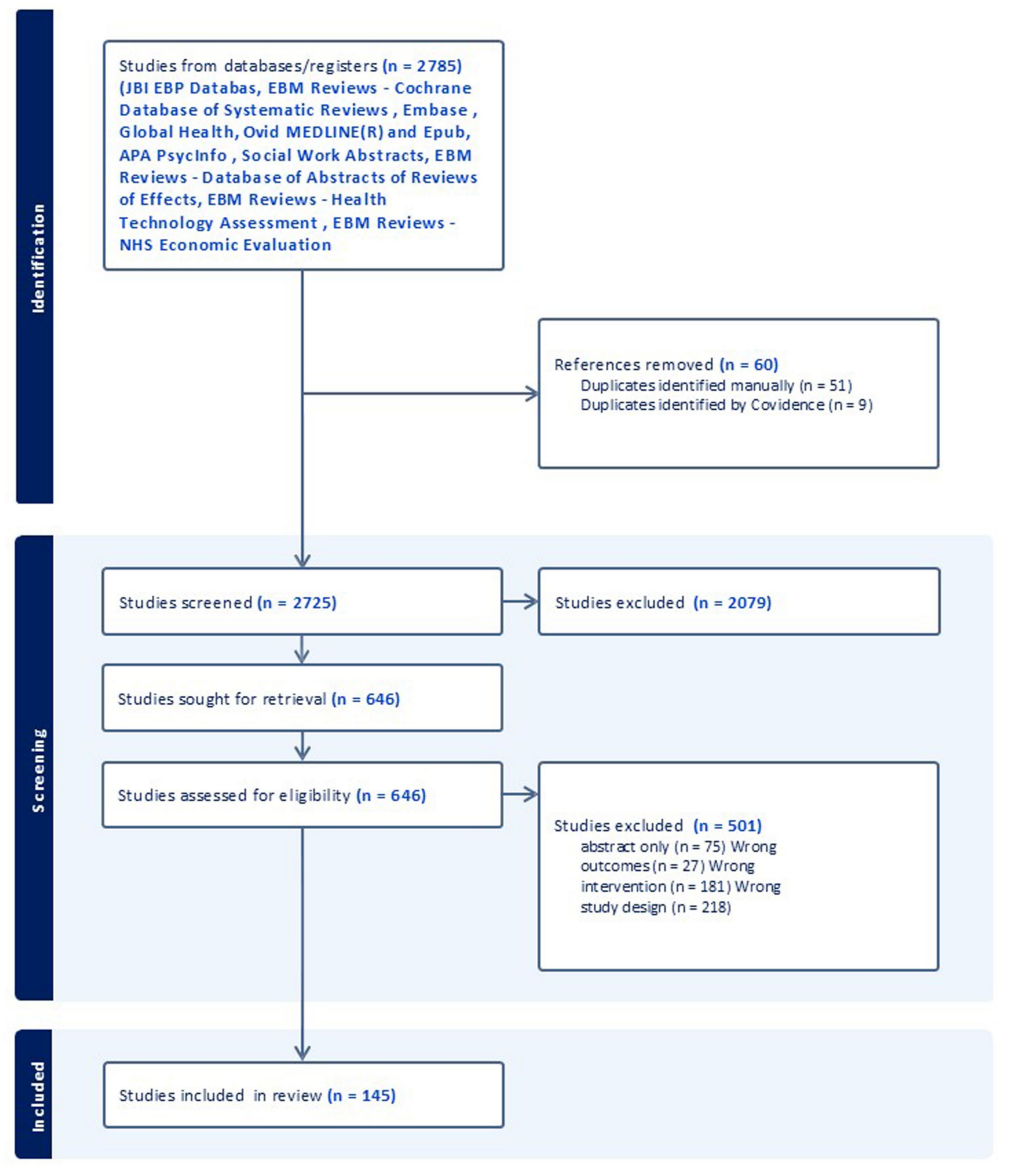
PRISMA chart according to Tricco et al. (18).

The PRISMA chart from the value-based healthcare scoping review outlines the study selection process (Figure 1). A total of 2,785 studies were identified from databases and registers. After removing 60 references due to duplication (51 manually and 9 using Covidence), 2,725 studies were screened. Out of these, 501 studies were excluded for various reasons, such as being abstracts only (75), having wrong outcomes (27), wrong interventions (181), or wrong study designs (218). A total of 2,725 studies were assessed for eligibility; However, 2079 studies were excluded throughout the process, leaving 646 for full text retrieval and 145 studies included in the final review. There were no ongoing studies, studies awaiting classification, or studies not retrieved.

### Study characteristics

**Figure 2:**
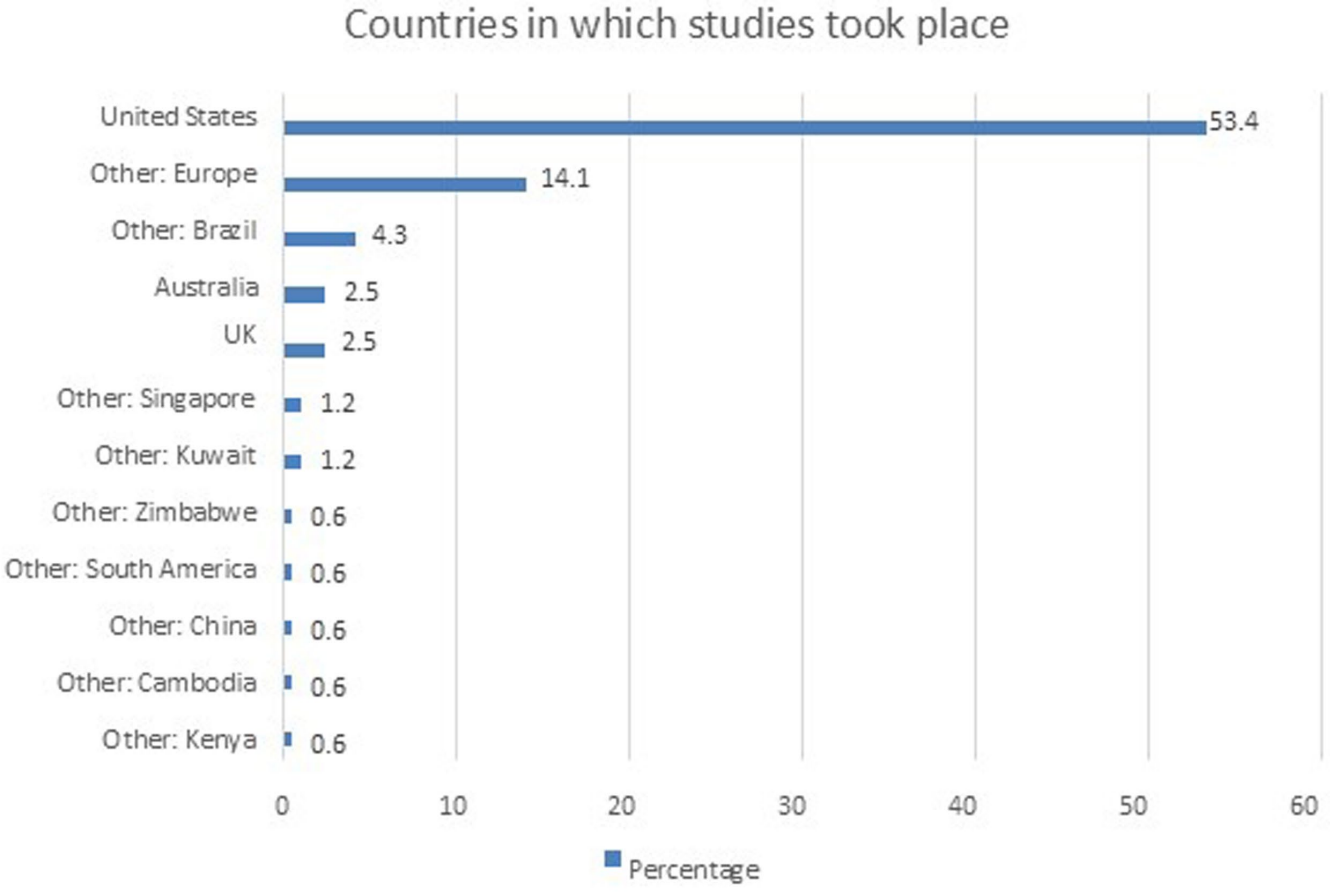
Countries in which studies were reported.

Figure 2 illustrates the distribution of studies conducted across various countries, with the United States overwhelmingly leading, contributing 87 studies, which represents 65% of the total. This significant dominance is followed by a collection of studies from European countries (17%), showing a broad engagement across multiple nations within the continent. Brazil contributes 5.2% of the studies, while other regions, such as the United Kingdom and Australia, each contribute 3.0%. Countries like Kuwait, Kenya, Singapore, Cambodia, China, and Zimbabwe represent smaller portions, each contributing between 0.7 and 1.5% of the total. This distribution indicates a concentration of research in the United States and Europe, with other regions playing smaller but still meaningful roles in global research efforts.

### Study design

**Figure 3:**
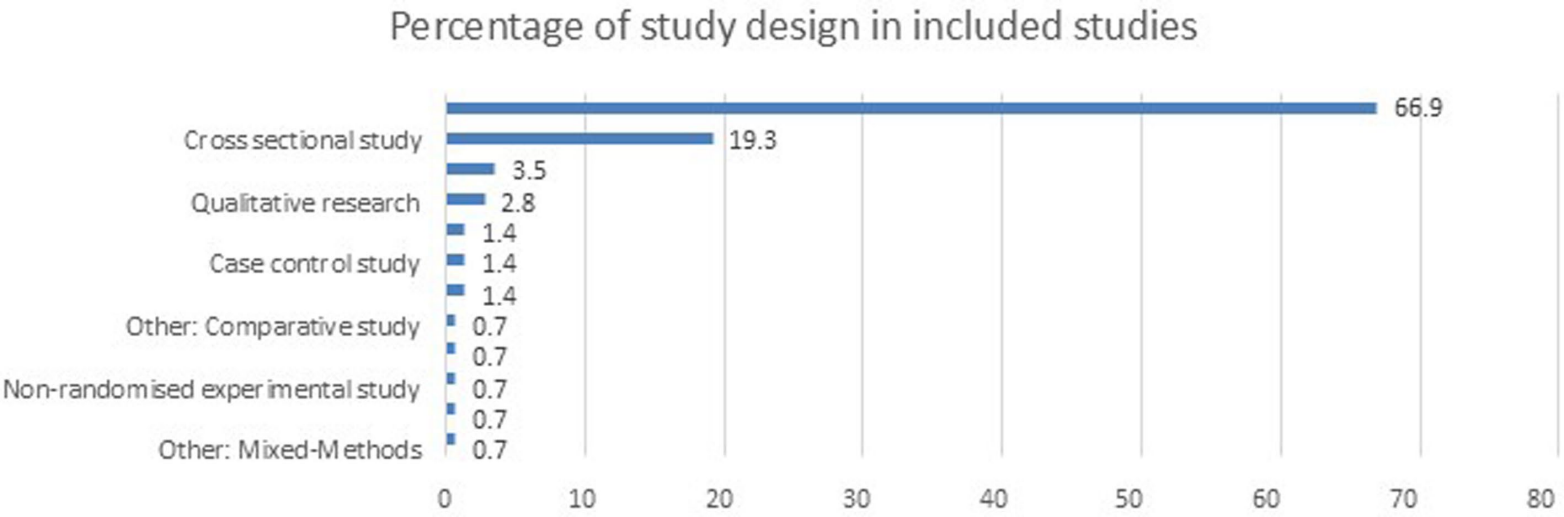
Study type included in the review.

Figure 3 illustrates the distribution of studies based on their design types, with cohort studies being the most predominant, comprising 67% of the total studies. Cross-sectional studies follow, accounting for 19.3%. Other study designs, such as case reports, case series, and case–control studies, each represent a much smaller portion, ranging from 1.4 to 3.4%. Additional study designs, including mixed methods, discrete choice experiments, qualitative research, and randomized controlled trials, contribute even smaller percentages, highlighting the dominance of cohort and cross-sectional designs in this dataset.

### Population

**Figure 4:**
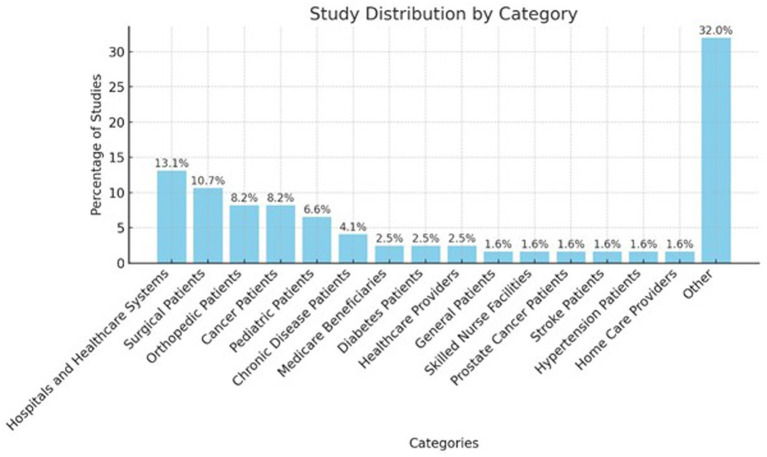
Study distribution by population types.

The bar chart (Figure 4) displays the distribution of studies by category, with “Hospitals and Healthcare Systems” accounting for 13.1% of the total studies and Surgical Patients following closely at 10.7%. Both Orthopedic Patients and Cancer Patients each represent 8.2%, while Pediatric Patients account for 6.6%. Chronic Disease Patients make up 4.1% of the studies. The remaining categories, such as Medicare Beneficiaries, Diabetes Patients, and Healthcare Providers, each represent 2.5%. Smaller groups like General Patients, Skilled Nurse Facilities, Prostate Cancer Patients, Stroke Patients, Hypertension Patients, and Home Care Providers each constitute 1.6% of the total studies. The “Other” category makes up the largest proportion, covering 32% of the studies.

The other category, encompasses 32% of the total, accounting for 39 studies. “Hospitals and Healthcare Systems” represent 13.1% with 16 studies, while Surgical Patients contribute to 10.7% of the total, with 13 studies. Both Orthopedic Patients and Cancer Patients are represented equally, each comprising 8.2% of the studies, equating to 10 studies each.

Pediatric Patients form 6.6% of the total, or eight studies, followed by Chronic Disease Patients at 4.1% with five studies. Medicare Beneficiaries, Diabetes Patients, and Healthcare Providers each account for 2.5%, or three studies. Other smaller categories, such as General Patients, Skilled Nurse Facilities, Prostate Cancer Patients, Stroke Patients, Hypertension Patients, and Home Care Providers, each represent 1.6% with two studies.

### Funding reimbursement models

**Figure 5:**
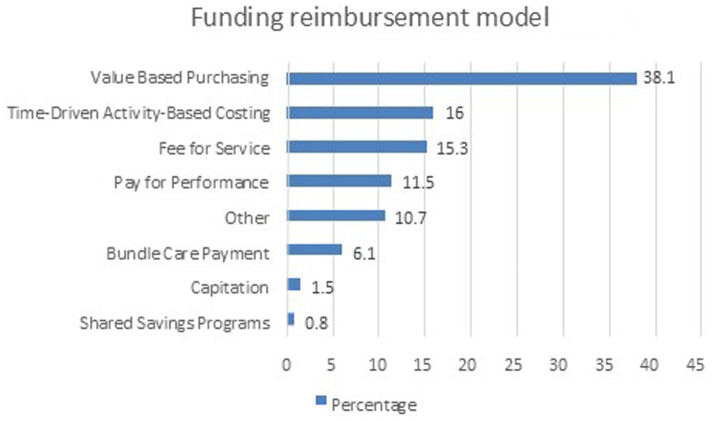
Distribution of funding models.

Only 99 (68%) studies reported a type of a funding model. Figure 5 represents the distribution of studies by funding models. The Value-Based Purchasing model is the most frequently used, accounting for 38.2% of the studies. Time-Driven Activity-Based Costing and Fee for Service models follow, representing 16.0 and 15.3% of the studies, respectively. The Other category accounts for 10.7%. Pay for Performance is used in 11.5% of the studies, while Bundle Care Payment appears in 6.1%. Less frequently used funding models include Capitation at 1.5% and Shared Savings Programs at 0.8%. This distribution shows a strong reliance on value-based purchasing, Time driven-activity-based models and fee for service, with some variations in performance-based and bundle care payment models.

### Models of care delivery

**Figure 6:**
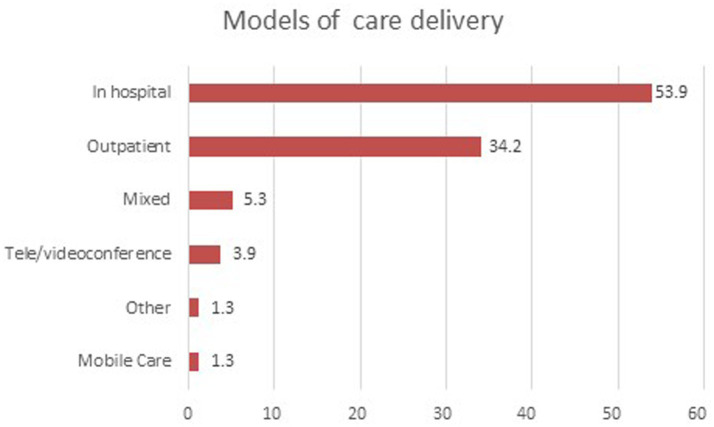
Models of care delivery.

Only a total of 133 studies reported on models of care. Figure 6 shows the distribution of studies by different models of care. The In-hospital model is the most prevalent, representing 53.9% of the studies. The Outpatient model follows, accounting for 34.2%. Mixed models of care are used in 5.3% of studies, while Tele/videoconference models make up 3.9%. Mobile care and other models each account for 1.3% of the studies. This distribution highlights the dominance of traditional in-hospital and outpatient care models, with emerging models like telehealth and mobile care playing a smaller but notable role.

### Outcome measures reported in the studies

The reported outcome measures were heterogenous across the studies and focus on various aspects of health care performance. These outcomes can be broadly categorized into the following areas:

1. Health Care Professionals’ Impact:
  - Many studies report the effects of specific healthcare interventions on health care professionals, such as efficiency, time, and resource utilization. For instance (19), examined the opportunity cost of intraoperative resident participation, highlighting significant time and cost differences when residents are involved in surgery (19). Similarly, Wong et al. (20, 21) focuses on financial impacts of dialysis initiation for professionals under value-based care contracts (20, 21).
2. Patient Outcomes:
  - Several studies focus on patient-centered outcomes, such as health outcomes achieved per dollar spent or improvements in quality of life. For example (22), aimed to develop outcome indicators for lung cancer, including patient-reported outcomes (PROMs) and survival metrics (22). Van Egdom et al. (22) measured how value-based health care improved breast cancer patients’ awareness of everyday function through PROMs (22).
3. Provider Outcomes:
  - Many studies also measure the impact of value-based healthcare models on providers. For instance (23), discussed the frustration of healthcare providers in adjusting to fixed reimbursements under the OrthoChoice program, revealing that many believed reimbursement was insufficient (23). Weiss et al. (24) highlighted that care management led to reductions in unnecessary hospital utilization, showing positive financial impacts for providers (24).
4. Cost Impact on Patients:
  - The cost implications for patients are another common outcome measure. Yu et al. (25) applied a time-driven activity-based costing (TDABC) approach to pediatric appendicitis cases, demonstrating a reduction in hospital stay costs by 11% (25). Similarly Thomas et al. (26), estimated cost savings for patients receiving compression garments through a redesigned procurement model, saving approximately £71 per patient (26).
5. Quality Impact on Patients:
  - Quality of care for patients is frequently assessed, with studies showing varied improvements in patient outcomes under value-based healthcare models. Teshale et al. (27) found that home health value-based purchasing programs resulted in improved quality care ratings for home health agencies (27). Sethi et al. (28) found significant differences in time spent with patients, linking these variations to quality improvements in post-operative care (28).
6. Cost Impact on Providers:
  - Many studies also examine the economic effects on healthcare providers. For example (29), analyzed reimbursement costs over 90 days for patients undergoing total elbow arthroplasty (TEA), identifying patient comorbidities and readmissions as major cost drivers (29). Reilly et al. (30) explored cost variability in total knee arthroplasty (TKA) and total hip arthroplasty (THA), revealing both cost and quality variations across hospitals (30).
7. Quality Impact on Providers:
  - In many instances, healthcare providers benefit from higher quality outcomes. Weiss et al. (24), for instance, reported that care coordination programs not only improved care quality but also offset program costs by reducing unnecessary hospitalizations (31). Orlandi et al. (32) also found a reduction in hospital wait times and cost per surgery while maintaining high-quality care in thoracic surgery (32).
8. Impact on Health Care Systems:
  - System-level outcomes are also measured, such as reduced hospital admissions or length of stay. Maki et al. (33) reported a significant decrease in non-urgent emergency medical services (EMS) visits, leading to substantial cost savings at the system level (33). Ramirez et al. (34) showed that specific performance scores helped categorize hospitals in a value-based care model, directly influencing hospital operational strategies (34).

### Barriers and facilitators

Both Barriers and facilitators of implementing value-based health care is shown in Table 1. The primary barriers to implementing value-based healthcare (VBHC) include insufficient funding, reliance on traditional fee-for-service models, and resistance from healthcare providers to adapt to VBHC concepts. Many healthcare systems lack the necessary IT infrastructure and data collection processes to support VBHC, as highlighted by challenges in capturing outcome measures and integrating patient-reported outcomes (35). Organizational structures are often not designed for multidisciplinary care, further hindering VBHC adoption (36, 37). Additionally, financial incentives are misaligned, with many providers still operating under fee-for-service reimbursement models, making it difficult to transition to value-based payment systems. Resistance from leadership and healthcare professionals, along with the time and resources required for data collection and analysis, also impede progress toward adopting VBHC (28, 38–41).

**Table 1:** Barriers and facilitators of implementing VBHC.

Barriers	
Insufficient funding and financial incentives	Many studies identify funding challenges as a significant barrier. Nguyen et al. (35) highlight insufficient funding as a hindrance to increasing service delivery, while Pestka et al. (37) noted difficulties in billing due to process barriers, particularly with patients who frequently change insurers. Adler-Milstein et al. (36) observed that voluntary participation in reform programs remains low due to complex requirements and a lack of financial incentive synergy.
Resistance to change among health professionals	Resistance from healthcare providers is frequently cited as a barrier. Daniels et al. (38) noted that healthcare professionals were resistant to adopting VBHC concepts, often due to a lack of leadership involvement and difficulty integrating VBHC into existing quality improvement systems. Sethi et al. (28) mentioned that TDABC (Time-Driven Activity-Based Costing) requires highly functional systems and a detailed understanding of patient care pathways, which can pose an operational challenge in many healthcare settings.
Lack of adequate data and IT infrastructure	Several studies mention that the absence of reliable data is a key barrier. Nilsson et al. (54) highlight the challenge of establishing outcome measures for treatments outside of surgery and the heavy reliance on IT contracts for patient care records. Dohmen et al. (39) emphasize the need for improved data registries and consistent data capturing, particularly for metrics that are not automatically captured, such as mortality rates.
Healthcare systems still oriented toward fee-for-service	Jain and Weiner (40) points out that the traditional fee-for-service model often stands in the way of VBHC implementation, as many systems are not designed to hold providers accountable for outcomes across the full continuum of care.
Organizational and structural barriers	Daniels et al. (38) mention that many organizations struggle to shift from a service-oriented to a disease-oriented model, and multidisciplinary care models are not always well supported. Structural and organizational resistance hinders the full integration of VBHC into healthcare systems

Facilitators	
Improved patient outcomes and cost efficiency	One of the strongest facilitators is the demonstrated improvement in patient outcomes and cost reduction. Wong et al. (20, 21) and Rocque et al. (43) both provide examples of VBHC models leading to significant cost savings for patients and health systems. Orlandi et al. (32) also demonstrate that focusing on outcomes rather than purely cost reduction helps prevent improper savings.
Implementation of patient-reported outcome measures (PROMs)	The use of PROMs is a recurring facilitator in VBHC implementation. Van Egdom et al. (22) and VanHooff et al. (22) report that the introduction of PROMs helps improve patient awareness of their health outcomes and facilitates the assessment of treatment value. Daniels et al. (38) also emphasize that seeing the results of PROMs encouraged health professionals to recognize the added value of VBHC.
Leadership and multidisciplinary care models	Strong leadership and multidisciplinary collaboration are important facilitators for successful VBHC implementation. Daniels et al. (38) highlight the role of inspirational medical leadership in increasing the belief in VBHC. Similarly, Wohlin et al. (23) suggest that close attention to purchaser regulations and strong provider involvement support successful value-based initiatives
Digital tools and data sharing	Digital platforms and transparent data sharing can significantly improve the implementation of VBHC. Misplon et al. (44) mention the success of digital follow-up in lung cancer care, where a high percentage of patients participated and reported ease of use. Dohmen et al. (39) also stress the importance of training providers to improve data collection and sharing, with transparency and benchmarking playing critical roles in motivating providers
Financial incentives for providers	In several studies, financial incentives and bonus structures were noted as key facilitators. Dohmen et al. (39) suggest that transparent benchmarking with financial rewards improves providers’ willingness to adopt VBHC. Grabowski et al. show that pay-for-performance models, though initially challenging, can drive providers to improve care quality over time

Facilitators of implementing value-based healthcare (VBHC) include the integration of patient-reported outcome measure (PROMs) and strong leadership support, both of which drive the success of VBHC initiatives (20, 21, 32, 42, 43). Inspirational medical leadership and multidisciplinary collaboration have been shown to enhance the implementation of VBHC by fostering a culture of continuous improvement and promoting buy-in from healthcare professionals (23, 38). Data transparency and benchmarking with other providers also motivate organizations to adopt VBHC, as providers see the value in performance comparisons and outcome improvements (39, 44). Additionally, the alignment of financial incentives with improved outcomes, as seen with bundled payments and value-based models, encourages healthcare systems to shift from volume to value-based care (39). The use of digital tools and telemedicine further facilitates the implementation of VBHC by improving access to care and optimizing costs while maintaining or improving patient outcomes (45).

## Discussion

The implementation of Value-Based Healthcare (VBHC) represents a transformative shift in how healthcare is delivered. VBHC focuses on maximizing patient outcomes relative to costs. This study mapped the characteristics of VBHC, and the challenges and enablers organizations encounter during implementation. Adopting Patient-Reported Outcome Measures (PROMs) ensures care alignment with patient priorities and improves care quality.

Furthermore, the study highlights the significance of data transparency and benchmarking in encouraging providers to improve their performance (46, 47). When healthcare organizations can compare their outcomes with peers and make data-driven decisions, it motivates continuous improvement. The use of financial incentives such as bundled payments and pay-for-performance models further aligns organizational goals with the core principles of VBHC by rewarding quality outcomes rather than volume-based services. This alignment is crucial for sustaining VBHC models over time, as it provides tangible benefits to both providers and patients (48, 49). The incorporation of digital tools, such as telemedicine platforms, was also identified as a facilitator, helping to reduce healthcare costs while maintaining or improving the quality of care. These tools can bridge gaps in access and offer a scalable solution for expanding the reach of VBHC initiatives.

The outcome measured reported by the included studies consistently focus on how value-based healthcare models impact cost, quality, and resource utilization for patients, providers, and health care systems. The implementation of value-based care models generally shows improvements in patient outcomes, a reduction in healthcare costs, and enhanced quality of care, although some studies highlight concerns regarding insufficient reimbursements for providers and variability in performance across different settings.

However, the study also highlights the barriers that must be overcome for successful VBHC adoption. A lack of sufficient funding, the persistence of fee-for-service models, and resistance from healthcare professionals to adapt to new processes pose significant challenges (35, 50). Addressing these barriers requires a strategic, system-wide commitment to integrating VBHC frameworks, including investments in health information technology and data collection infrastructure. This study demonstrates that while the way to fully integrated VBHC systems is complex, the potential benefits in terms of improved patient outcomes, cost savings, and healthcare quality make it a worthwhile Endeavor for health systems worldwide. The findings provide critical insights for policymakers, healthcare providers, and institutions seeking to implement or refine VBHC models in diverse healthcare settings (51).

The results of this study closely aligns by a recent review published by Fernández-Salido et al. (2), where the authors examines the current state of Value-Based Healthcare (VBHC) and its key elements for successful implementation (2). The review finds consensus on the definition of VBHC, which focuses on improving health outcomes relative to the costs of care, but there is variability in how different studies interpret and implement the model. Key elements frequently mentioned include strong leadership, patient involvement, integrated care units, standardizing outcome measured, and utilizing updated IT systems. However, the lack of a unified understanding of VBHC creates differences in how these elements are applied, leading to variability in outcomes. The study suggests that a more consistent, standardized approach is needed to fully realize the potential of VBHC and ensure it improves healthcare efficiency and sustainability. Positive outcomes identified include improved cooperation among professionals, better patient follow-up, and more effective measurement of outcomes, but the study highlights the need for further research to address gaps in implementation and scalability (2).

Our current study differs from a few recently published reviews by De Mattia et al. (52) and Van Staalduinen et al. and in several keyways, particularly in how Value-Based Healthcare (VBHC) is conceptualized, implemented, and evaluated across different healthcare systems (52, 53).

First, while this current review presents VBHC as a well-established framework aimed at improving patient outcomes while managing costs, the recently published work lacked a unified conceptualization of VBHC. For instance, Van Staalduinen et al. emphasize that many healthcare organizations implement only select components of VBHC, such as outcome measurement and integrated practice units, rather than adopting a comprehensive approach. This variability in definitions and application makes it difficult to compare studies and assess VBHC’s true impact (53). In contrast, our review assumes a more standardized understanding of VBHC, outlining its facilitators and barriers without fully acknowledging the inconsistencies in how it is interpreted across different settings.

Secondly, this current review broadly discusses the facilitators of VBHC, such as strong leadership, data infrastructure, and financial incentives, while also detailing challenges like resistance to change and funding constraints. However, the article by De Mattia et al. (52) provides a more structured analysis of VBHC implementation strategies, categorizing them into macro (government and policy), meso (hospital administration), and micro (healthcare provider) levels. Their findings suggest that hospitals play a central role in transitioning to VBHC but cannot achieve success in isolation. A comprehensive, multi-level approach is necessary, involving government support, appropriate reimbursement models, and organizational restructuring. The current review acknowledges these factors but does not clearly differentiate between policy-level and provider-level strategies.

Another key difference lies in the discussion of outcome measurement and effectiveness. This current review acknowledges that VBHC can lead to cost reductions and improved clinical outcomes but highlights the heterogeneous nature of reported results. Fernández-Salido et al. (2), however, stress that many studies lack long-term evaluations of VBHC’s effectiveness. The authors argue that while VBHC holds promise, its impact remains difficult to assess due to inconsistencies in measuring patient outcomes, financial benefits, and healthcare quality. Their review also highlights the importance of standardizing outcome measures.

The limitations of this study are important to acknowledge as they may affect the generalizability and applicability of the findings. Firstly, the heterogeneity of the included studies presents a challenge. The studies examined different populations, healthcare settings, and value-based healthcare models, making it difficult to standardize comparisons across diverse healthcare systems. This variability may limit the ability to draw uniform conclusions about the effectiveness of VBHC across all contexts. Additionally, many of the studies were observational in nature, which introduces the possibility of selection bias and limits the ability to establish causal relationships between the implementation of VBHC and the reported outcomes.

Another limitation is that value is a concept closed linked to cultural contexts. However, the included studies in this scoping review are dominated by those from the US and the European countries. The two largest populated countries, India and China, contributed little in the included literature. This may be associated with the language restriction and the lack of resources in those countries in exploring and testing new funding and care models based on VBHC, although VBHC is even more important for resource-restraint countries.

Another significant limitation is the reliance on self-reported data from healthcare providers and organizations. This data may be subject to reporting bias, where participants may overestimate the success of VBHC implementations due to social desirability or institutional pressure. Moreover, many of the studies lacked long-term follow-up, which is essential to fully understanding the sustainability of VBHC models over time. Short-term gains in cost reduction or quality improvements may not translate into long-term success, and without extended observation periods, the durability of VBHC benefits remains unclear.

Lastly, the study was limited by the availability of comprehensive data on the financial and clinical outcomes of VBHC initiatives. In many cases, the studies provided incomplete financial data or focused primarily on process measured rather than patient-centered outcomes. This restricts the ability to evaluate the true economic and quality-of-care impacts of VBHC models, particularly in terms of long-term cost savings and improvements in patient health outcomes. More robust data collection and consistent outcome measured are needed to address these gaps and provide a more thorough assessment of VBHC effectiveness.

## Conclusion

While the implementation of Value-Based Health Care (VBHC) holds promise for improving healthcare quality and cost-efficiency, the current evidence highlights several challenges and limitations that must be addressed. The variability in healthcare settings, models, and populations makes it difficult to generalize the results, and the reliance on short-term, self-reported data further complicates the assessment of VBHC’s long-term impact. Although there are examples of successful cost reductions and quality improvements, the lack of consistent financial and clinical outcome data hinders a comprehensive evaluation of VBHC’s effectiveness. To fully realize the potential of VBHC, more standardized approaches to measuring outcomes, better data collection, and long-term studies are necessary to ensure sustainability and scalability across diverse healthcare systems.
