# Supplementary material for: Implementing value-based healthcare: a scoping review of key elements, outcomes, and challenges for sustainable healthcare systems
Source: Front Public Health. 2025 Apr 9;13:1514098. doi: 10.3389/fpubh.2025.1514098 (PMC12014573; doi:10.3389/fpubh.2025.1514098)
Supplement: Supplementary file 1 [file Table_1.docx]

**Appendix 1**

**Search strategy**

Database: JBI EBP Database <Current to November 08, 2023>, EBM Reviews - Cochrane Database of Systematic Reviews <2005 to November 15, 2023>, Embase Classic+Embase <1947 to 2023 November 17>, Global Health <1910 to 2023 Week 46>, Ovid MEDLINE(R) and Epub Ahead of Print, In-Process, In-Data-Review & Other Non-Indexed Citations, Daily and Versions <1946 to November 17, 2023>, APA PsycInfo <1806 to November Week 2 2023>, Social Work Abstracts <1968 to September 2023>, EBM Reviews - Database of Abstracts of Reviews of Effects <1st Quarter 2016>, EBM Reviews - Health Technology Assessment <4th Quarter 2016>, EBM Reviews - NHS Economic Evaluation Database <1st Quarter 2016>

Search Strategy:

1 exp Value-Based Health Care/

2 "value based health care".mp. [mp=tx, hw, sw, ti, ab, kw, ct, tn, ot, dm, mf, dv, kf, fx, dq, cw, bt, nm, ox, px, rx, ui, sy, ux, mx, tc, id, tm, sh]

3 "value based health system*".mp. [mp=tx, hw, sw, ti, ab, kw, ct, tn, ot, dm, mf, dv, kf, fx, dq, cw, bt, nm, ox, px, rx, ui, sy, ux, mx, tc, id, tm, sh]

4 "value based healthcare".mp. [mp=tx, hw, sw, ti, ab, kw, ct, tn, ot, dm, mf, dv, kf, fx, dq, cw, bt, nm, ox, px, rx, ui, sy, ux, mx, tc, id, tm, sh]

5 "value based care model*".mp. [mp=tx, hw, sw, ti, ab, kw, ct, tn, ot, dm, mf, dv, kf, fx, dq, cw, bt, nm, ox, px, rx, ui, sy, ux, mx, tc, id, tm, sh]

6 "Value based health care framework*".mp. [mp=tx, hw, sw, ti, ab, kw, ct, tn, ot, dm, mf, dv, kf, fx, dq, cw, bt, nm, ox, px, rx, ui, sy, ux, mx, tc, id, tm, sh]

7 1 or 2 or 3 or 4 or 5 or 6

8 limit 7 to english language [Limit not valid in JBI EBP Database,CDSR,SWAB,DARE; records were retained]

9 limit 8 to human [Limit not valid in JBI EBP Database,CDSR,Global Health,SWAB,DARE; records were retained]

10 limit 9 to yr="2000 -Current" [Limit not valid in DARE; records were retained]

**Appendix E:** Outcomes measures

| Study ID | Title | Cost outcome- Patient | Quality outcome- Patient | Cost outcome- Provider | Quality outcome- Provider | Cost outcome- Health Professional | Quality outcome- Health Professional |
| --- | --- | --- | --- | --- | --- | --- | --- |
| Zhu et al. 2019 | The Cost to Attending Surgeons of Resident Involvement in Academic Hand Surgery |  |  | There was a statistically significant increase of 24.3 minutes (P < 0.001) in the mean operation time with a resident present as compared with those without.  Moreover, Relative Value Units per hour in resident cases was significantly lower by 2.97 RVU per hour or 21%  Using the late 2018 Medicare physician conversion factor of US $33.9996, the opportunity cost to attending physicians is US $159.20 per case. |  |  |  |
| Zhao et al. 2022 | Telehealth and hospital performance: Does it matter? |  |  | The more telehealth services within the hospital the greater the cost reduction for care | No significant differences noted in the domains of clinical care, person and community engagement and safety |  |  |
| Zhang & Cowling 2023 | Association of Participation in a Value-Based Insurance Design Program With Health Care Spending and Utilization | The program encouraged decreased inpatient and surgical admissions that influence Out of Pocket Payments | The program encourage higher utilisation of primary care physicians. | |  |  |  |
| Yuhua et al. 2017 | Value-based payment in implementing evidence-based care: the  Mental Health Integration Program in Washington State |  |  |  | Estimated marginal effects of VBP on fidelity ranged from 9% to 30% of the level of  fidelity had there been no exposure to VBP (p<0.05 for every fidelity measure). Improvement in  fidelity in response to VBP was greater among providers with a larger patient panel and among providers with a lower level of fidelity at baseline. Exposure to VBP was associated with an  adjusted hazard ratio of 1.45 (95% CI: 1.04â€“2.03) for achieving clinically significant improvement  in depression |  |  |
| Yu et al. 2017 | Time-driven activity-based costing: A dynamic value assessment model in pediatric appendicitis |  |  | The post-intervention TDABC model featured 6 phases of care, 33 processes, and 19 personnel types. Our interventions reduced duration and costs in the emergency department (âˆ’41 min, âˆ’$23) and pre-operative floor (âˆ’57 min, âˆ’$18). While post-anesthesia care unit duration and costs increased (+224 min, +$41), the same-day discharge protocol eliminated post-operative floor costs (âˆ’$306). Our model incorporating all three interventions reduced total direct costs by 11% ($2753.39 to $2447.68) and duration of hospitalization by 51% (1984 min to 966 min). |  |  |  |
| Wong 2023 | Dialysis Costs for a Health System Participating in Value-Based Care |  |  | 260 crash starts, 130 optimal starts, and 105 suboptimal starts. Median predialysis 12-month cost was $67,059 for crash starts, $17,891 for optimal starts, and $7633 for suboptimal starts (P <.001). Median postdialysis 12-month cost was $71,992 for crash starts, $55,427 for optimal starts, and $72,032 for suboptimal starts (P = .001). Predialysis inpatient admission per 1000 beneficiaries was 1236 per 1000 for crash starts vs 273 per 1000 for optimal starts and 170 per 1000 for suboptimal starts (P <.001). Postdialysis inpatient admission for crash starts was 853 per 1000 vs 291 per 1000 for optimal starts and 184 per 1000 for suboptimal starts (P <.001) |  |  |  |
| Wolosin et al. 2012 | Nursing Care, Inpatient Satisfaction, and Value-Based Purchasing |  |  |  | Each 1-point increase in the nursing domain score increased the odds of achieving an HCAHPS top-box score by 4.9%. Increases in other domain scores also affected top-box odds, although to a smaller extent |  |  |
| Wohlin et al. 2021 | As predicted by theory: choice and competition in a publicly funded and regulated regional health system yield improved access and cost control |  |  | Patients entered OrthoChoice through referrals from primary care. Providers expressed frustration over the quality of the referrals they received and that they had to rely on primary care to conduct the initial assessment as they were punished financially if a referred patient did not meet indications for surgery.  Providers unanimously stated that the fixed reimburse- ment for OrthoChoice patients was too low, and subse- quent price adjustments were insufficient. Provider representatives claimed considerably higher unit costs, although no provider could provide detailed information on how those estimates had been calculated | A majority of providers were positive to the introduction of OrthoChoice and defined it as a manifestation of patient-centredness  All providers reiterated that they paid close attention to the regulations set by the purchaser |  |  |
| Wittlieb-Weber et al. 2015 | Pediatric Versus Adult Cardiomyopathy and Heart Failure-Related Hospitalizations: A Value-Based Analysis | Charges were greater for pediatric hospitalizations, both overall ($116,483 vs $40,662 ) and for all years evaluated | Overall mortality was greater for pediatric hospitalizations , although it decreased over time for both pediatric and adult hospitalizations |  | Pediatric cardiomyopathy and heart failure hospitalizations were significantly longer than adult hospitalizations |  |  |
| Wickman et al. 2022 | Influence of medical comorbidity and surgical indication on total elbow arthroplasty cost of care |  |  | The mean reimbursement cost of the surgical encounter ($13,393) did not differ significantly based on patient factors. The mean reimbursement cost of the 89-day postoperative period ($4232) differed significantly when stratified by surgical indication or by medical comorbidity. The total 90-day reimbursement cost ($16,982 ) differed significantly when stratified by surgical indication or by medical comorbidity , with the indication of acute fracture ($18,870) and the comorbidity of chronic pulmonary disease ($19,194) showing the highest total 90-day cost. Inpatient costs related to readmissions represented 38% of the total reimbursement cost. The overall readmission rate was 5.0%, and the mean readmission cost was $16,296 |  |  |  |
| Weiss et al. 2019 | Effective Care Management for  Children With Special Health  Care Needs in the Era of  Value-Based Payment |  | Significant reduction in ED and inpatient utilisation | While the costs of the program exceeded  the amount budgeted within a total capitation funds flow, the financial benefits from cost savings more than adequately covered the program costs. | Significant reduction in ED and inpatient utilisation |  |  |
| VanHooff et al. 2017 | Care for Outcomesâ€™: systematic  development of a set of outcome  indicators to improve patient-relevant  outcomes for patients with lung cancer |  |  |  | The resulting set of indicators consists of only six variables from three different  outcome hierarchy levels: survival (mortality and median  survival after diagnosis); degree of recovery (treatment  result after resection and QoL) and process of recovery  (complications after resection and side effects after  radiotherapy or chemotherapy). These indicators, tested  for their feasibility and discriminative aspect to patient  outcomes, differ from most quality indicators nationwide,  which focus on the process and structure of care or on  only a part of clinical care for patients with lung cancer. |  |  |
| van Egdom et al. 2019 | Implementation of Value Based Breast Cancer Care |  | Inclusion of Patient Reported Outcomes (Measures) assisted patients in becoming more aware of everyday function and contributed positively to their treatment. | |  |  | Care providers at our institute reported similar benefits, additionally stating that with the use of PROMs a more complete view about the provided care can be obtained.  Clinicians found PRO data useful and not disruptive to their practice |
| vanDeena et al. 2017 | The impact of value-based healthcare for inflammatory bowel diseases on healthcare utilization: a pilot study |  | Consistently observed trends toward fewer ED visits, hospitalizations, surgeries, upper endoscopies, and most imaging tests, as well as less long-term corticosteroid use | Costs decreased by $221 per patient compared to $73 in the non VBHC group |  |  |  |
| Trenaman et al. 2023 | Medicare Beneficiariesâ€™ Perspectives on the Quality of Hospital Care and Their Implications for Value-Based Payment |  | A hospitalâ€™s performance on clinical outcomes was most highly valued by beneficiaries (49%), followed by safety (22%), patient experience (21%), and efficiency (8%) | Nearly double the number of hospitals would see a payment reduction when using beneficiary value weights than would see an increase (1830 vs 922 hospitals).  Overall, those seeing a net reduction were more likely to be in less populated areas and have fewer hospital beds and admissions and serve less-complex patients, as measured by CMI. |  |  |  |
| Tragl et al. 2023 | Who counts when health counts? A case-study of multi-stakeholder initiative to promote value-creation in Swedish healthcare |  |  |  | The multiple stakeholder innovation network described in this case was successful in reaching the intended deliverables in the form of eight benchmarking reports that applied sophisticated case-mix adjustment algorithms and an analysis platform for managing data and visualising information. |  |  |
| Tozzi et al. 2023 | Using big data and Population Health  Management to assess care and costs  for patients with severe mental disorders  and move toward a value-based payment  system |  |  | Annual mean costs per patient across regions was â‚¬3,925, ranging from â‚¬3,101 to  â‚¬6,501 in the three regions. Some 70% of total costs were for MH services and medications, 37% incurred in dedicated  mental health facilities, 33% for MH services and medications noted in physical healthcare databases, and 30% for other conditions |  |  |  |
| Thomas et al. 2021 | Changing the process of prescribing to  procuring lymphoedema compression  garments: a service evaluation | hen all costs are considered, there is an  individual patient cost saving of Â£71.10 (SD Â£40.80)  when procuring is compared to prescription | There are differences between  the timing and quality of care the patients receive  comparing the prescription to procuring route.   the risk of error with the additional  steps in prescribing will likely further impact  concordance and patient outcomes |  |  |  |  |
| Teshale et al. 2021 | Early Effects of Home Health Value-Based  Purchasing on Quality Star Ratings |  | We also found similar  proportions of agencies showed increases in patient experience star ratings in both the HHVBP and non-HHVBP  groups |  | 30.7% of agencies in HHVBP were in the 4- or 4.5-star group, and from January to December 2016, 34.9% were in the 4- or 4.5-star group. By comparison, in the non-HHVBP group, 24.1% of agencies  were in the 4- or 4.5-star rating group in 2015, and 24.63%  were in the 4- or 4.5-star rating group in 2016 |  |  |
| Tavengwa et al. 2023 | A pilot study to explore societal, patient, and public authority perception on â€˜Value- Added Taxâ€™ system for healthcare financing in Zimbabwe: A case for cancer treatment | The general public and cancer patients perceived cancer treatment as generally unaffordable and showed readiness to pay for a cancer levy through a value added tax |  | Public authorities also perceived the VAT system as an appropriate programme for health care financing |  |  |  |
| Tanet al. 2016 | Using Quality Improvement Methods and Time-Driven Activity- Based Costing to Improve Value- Based Cancer Care Delivery at a Cancer Genetics Clinic |  |  | Cost calculations using TDABC showed that direct personnel costs of the patient care cycle were lowered by 18%. | increase in clinic capacity of 350%, surpassing our original goal of 100%  For patients who subsequently underwent genetic testing during the study period, the quarterly average time from sample acquisition to dis- closure of results also decreased from 91 days (range, 56 to 126 days) in April 2014 to 42 days (range, 27 to 56 days) in April 2015 |  |  |
| Stone et al. 2022 | Understanding the Economic Impact of an Essential  Service: Applying Time-Driven Activity-Based Costing  to the Hospital Airway Response Team |  |  | The total staffing costs were estimated to be $218,601  for the 2-year study period when considering the time  estimates alone  The revenue generated by the airway team through  the CPT code 31500 was $207,181 over the period of 2  years  If the single attending  only responded to Stat pages and performed intuba- tionsâ€”in essence, an airway team that provided the  minimal serviceâ€”the 2-year staffing cost would be  $177,406 for the 2-year period |  |  |  |
| Stanberry et al. 2021 | Using the MEAT VBP Framework to analyse and understand the value of surgical gloves: an explanatory case study |  |  | ten cost criteria and eight outcome criteria were identified with which the value of surgical gloves can be analysed and understood. For each of these criteria we propose definitions and value impact metrics that decision- makers can use during a procurement exercise to describe, quantify and compare glove value |  |  |  |
| Sreeram et al. 2021 | Patient-Reported Outcome Measures and Clinical Outcomes in Children with Foregut Anomalies |  | In children with CDH and EA, lower cognition was significantly associated with lower self-reported HS. | |  |  |  |
| Spaulding et al. 2021 | Race to the Top of the Hospital Value-Based  Purchasing Program |  |  | organizations that were expected to have robust and rigid resources were unable to score  in the superior category consistently. In addition, organizations were unable to consistently  perform positively over time because of changes in the HVBP program measurement and  the required organizational responses. |  |  |  |
| Spaulding et al. 2020 | Do MagnetÂ®-Designated Hospitals Perform Better on Medicare's Value-Based Purchasing Program? |  |  |  | Magnet-designated hospitals were associ- ated with higher total performance, process of care and patient experience of care scores, and lower efficiency score   No association was identified between the length of time hospitals have been Magnet designated |  |  |
| Spaulding et al. 2018 | Hospital Value-Based Purchasing Performance: Do Organizational and Market Characteristics Matter? |  |  |  | statistically significant relationship between level of centralization and each of the HVBP domains  Independent hospital systems are associated with lower scores than moderately centralized health systems across all four HVBP scores  For every 1 unit increase in the Medicaid percentage of the hospital, a 12.59-point decrease occurs in the TPS |  |  |
| Snow 2023 | Managing Total Knee Replacement Under Value- Based Payments |  |  | Decreased cost on the provider | Development of process changes relevant to outcomes measures |  |  |
| Sethi et al. 2021 | Utilizing Lean Methodology and Time-Driven Activity-Based Costing Together |  |  |  | Total time spent with patients was significantly higher for patients released on postoperative day 2 compared with postoperative day 1  The amount of time spent with patients did not differ during the preoperative or PACU phases for any of the personnel  significant differences in the time spent with patients during the surgery phase |  |  |
| Sethi et al. 2022 | Combining time-driven activity-based costing and lean  methodology: an initial study of single-level lumbar fusion  surgery to assess value-based healthcare in patients  undergoing spine surgery |  |  |  | Comparisons indicated that patients discharged on POD 1 spent significantly less time in surgery (mean 120.00, SD 58.35 minutes) compared to those released on POD 2 (mean 191.54,  SD 80.88 minutes; p = 0.004), and POD 3 (mean 242.82,  SD 92.29 minutes; p < 0.001). |  |  |
| Schoonbeek et al 2021 | Determinants of delay in the head and neck oncology care  pathway: The next step in value-based health care |  | Patients with a oropharyngeal or hypopharyngeal tumour  more frequently belonged to the delayed group than patients with oral cavity of laryngeal cancer    Other risk factors for delay  were advanced tumour stage and (chemo)radiation as treatment modality |  | Geriatric assessment showed a significantly higher number of patients with risk of malnutrition in the delayed group   total of 57 patients (29.7%) started treatment within 30 days of histopathological confirmation of malignant disease ( |  |  |
| Sanchez-Gavilan et al. 2022 | Added value of patientâ€‘reported outcome  measures (PROMs) after an acute stroke  and early predictors of 90 days PROMs |  | At 3 months, 48.7% patients reported unfavourable results in PHY-PROMIS 39.3% showed affected mental outcomes  in M-PROMIS, 30.8% presented with HADS-detected  depression and 21.5% with anxiety in HADS sub-scale.  Only PHY-PROMIS showed a significant statisti- cal improvement from day 7 to 3 months (p < 0.01)  and OHS from day 7 to 1 year (p = 0.03); the remain- ing PROMs did not statistically improve along the  follow-up. From all patients reporting depression on  HADS at 3 months, 54.1% continued to report depression after 1 year. |  |  |  |  |
| Salampessy et al. 2018 | The effect of cost-sharing design characteristics on use of health care recommended by the treating physician; a discrete choice experiment | Patients preferred afterwards billing of cost-sharing payments instead of at point of care by health insurers Most Patients are unaware of co-payments and prices associated with receiving care. | Less complex cost-sharing programs improved adherence to recommended care. |  |  |  |  |
| Ryan et al. 2017 | Changes in Hospital Quality Associated  with Hospital Value-Based Purchasing |  |  |  | HVBP did not result in improvements in clinical process or patient experience or a significant reduction in mortality during the first 4 years |  |  |
| Russell et al.2020 | Longitudinal Trends in Costs for Hospitalizations at Childrenâ€™s Hospitals |  |  | The room category was the most consistent driver of cost growth of all service lines examined. Mean annual increases in this category were $136 for medical and$160 for surgical aggregated service lines and ranged from $51 to $282 across the top 6 individual service lines.  The 3 medical service lines with the highest rate of annual change in adjusted cost per discharge were oncology (3.5%), reproductive services (2.9%), and orthopedicsâ€“medical (2.8%)  The 3 surgical service lines with the highest rate of annual change in adjusted cost per discharge were solid organ transplant (3.7%), ophthalmology (3.3%), and otolaryngology (2.9% |  |  |  |
| Rosenthal et al. 2007 | Employersâ€™ Use of Value-Based Purchasing Strategies |  | many large employers are not using their purchasing power with health plans and providers to improve the quality of health care received by their employees  Only a minority,  reported working with health plans to select quality improvement projects.   Finally, roughly one-fifth of employers reported participation in a local or regional health care purchasing coalition. |  |  |  |  |
| Rocque et al. 2017 | Resource Use and Medicare Costs During Lay Navigation for Geriatric Patients With Cancer |  |  | Costs per patient declined $728.29USD per quarter faster in the navigation group than the comparison group | Decreased resource use with the navigated group |  |  |
| Robards et al. 2022 | A value-based approach to prostate cancer image-guidance in a regional  radiation therapy centre: a cost-minimisation analysis |  |  | The health service saved an average of AU$ 361 per patient | There was no significant difference in fiducial marker position pre- and post- implementation |  |  |
| Riley et al. 2019 | A framework for oral health care value-based payment approaches |  | Increased quality of care under VBF | |  |  |  |
| Reilly et al. 2020 | Creating a Value Dashboard for Orthopaedic Surgical Procedures |  |  | TKA direct cost ranged from 10% below to 7% above the institutional benchmark, and the TKA value ranged from 7% below to 12% above the institutional benchmark   the THA direct cost ranged from 4% below to 3% above the institutional benchmark, and the THA value ranged from 12% below to 7% above the institutional benchmark | overall quality of the TKAs ranged from 16% below to 5% above the institutional benchmark  THA quality ranged from 10% below to 1% above the institutional benchmark |  |  |
| Reif et al. 2021 | Effectiveness of value-based purchasing for substance use treatment engagement and retention |  |  |  | There was no impact of the incentive-based contract on the treatment engagement, retention, and completion measures.  Heterogeneity of the client population (i.e., case mix) makes it hard for a program to change retention across the full caseload |  |  |
| Rangnekar et al. 2015 | The Relationship Between Hospital  Value-Based Purchasing Program  Scores and Hospital Bond Ratings |  |  | 15% had been assigned a bond rating of Aa,  and 46% had been assigned an A rating  Inclusion of VBP scores in the methodology  used to determine hospital bond ratings is likely to affect hospital bond ratings in  the near term. |  |  |  |
| Ramirez & Brennan 2020 | Using the value-based care paradigm to compare physical therapy access to care models in cervical spine radiculopathy: a case report | Direct access physical therapy saved the patient and third-party payer $434.30 and $3264.75 respective | A 5Ã—â€™s higher efficiency per visit and a 6.2Ã—â€™s higher value in reducing disability was demonstrated when the patient accessed physical therapy directly. |  | Physician referral and direct access entry pathways demonstrated neck disability index improvements of 6% and 16%, respectively. |  |  |
| Ramirez et al. 2016 | Physician-Owned Surgical Hospitals Outperform Other Hospitals in Medicare Value-Based Purchasing Program |  |  |  | Estimated mean total performance scores for physician-owned surgical hospitals and Kaiser Permanente were significantly higher compared with all remaining hospitals |  |  |
| Qi 2020 | Performance and Penalties in Year 1 of the Skilled Nursing Facility Value-Based Purchasing Program |  |  | 72% (10 436) of SNFs were penalized; 21% (2996) received the maximum penalty of 1.98%. | rural SNFs were less likely to be penalized  SNFs with lower nurse staffing had higher odds of penalties  small SNFs were more likely to be penalized  nonprofit and government-owned SNFs had lower odds of penalties |  |  |
| Pestka et al. 2020 | Community pharmacists' perceptions  of acceptability, appropriateness, and  feasibility of a value-based care model for  comprehensive medication management |  |  | Managers disagreed with the funding model being utilised |  |  | CMM does increase value of care delivered to patients, and drives pharmacists to perform higher. |
| Perera et al. 2022 | The Effects of Home Health Value-Based  Purchasing on Home Health Care Quality  in For-Profit and Nonprofit Agencies: A  Comparative Interrupted Time-Series  Analysis, 2012â€“2018 |  | There were no improvements in Patient Experience under  the HHVBP model among for-profit HHAs, but there were  slight improvements (p < .05) in nonprofit agencies during  the post-implementation period. |  | increase in Care Quality among for-profit HHAs in  2016,   nonprofit agencies,  the HHVBP effect upon Care Quality was not significant  nonprofit and hospital-based agencies cared for greater  numbers of high-risk patients compared with for-profit and  freestanding agencie |  |  |
| Parra et al. 2017 | Assessing value-based health care delivery for haemodialysis |  |  |  | Multi-criteria methodology can be used to measure quality |  |  |
| Panchal et al. 2023 | The implementation of value-based frameworks,  clinical care pathways, and alternative payment  models for cancer care in the United States |  | Value frameworks are not patient-oriented | |  |  | Larger physician buy in when provider driven not payer driven |
| Orlandi et al. 2023 | Multi-level analysis and evaluation of organizational improvements  in thoracic surgery according to a Value-Based HealthCare  approach |  |  | Hospitalisation costs decreased from 3.006 euros in 2017 to 2.050 euros in 2019 | Hospital stays and wait times for surgery decreased |  |  |
| Okeke et al. 2021 | Implementing Value-Based Primary Care Delivery in Federally Qualified Health Centers |  |  | The FPCC 3-year investment of $4.4MM yielded a cumulative cost savings of $19.4MM | There was a 35% decrease in ED utilization per 1000 and a 2.1% decrease in percent ED Non-Sudden and Serious diagnosis. For the IP targets, there was an 11% decrease in admits per 1000, and slight increase (about 2%) in IP days/1000 and average length of stay (ALOS).  The strategies/tactics that were funded primarily by FPCC payments were mainly aligned with new member access, episodic care management, and addressing social barriers |  |  |
| O'Donnell et al. 2023 | Coaching to Bedside Shift Report and Its Correlation to Hospital Consumer Assessment of Healthcare Providers and Systems and Value-Based Purchasing Dimension Scores |  |  | Value-based purchasing points and percentages increased over 2017-2020, potentially leading to lower CMS penalty claims over the period the BSR was implemented | Coaching to BSR had a significant impact on top- and bottom-box â€œrate the hospitalâ€ HCAHPS scores at a system and hospital level. |  |  |
| Nycz et al. 2020 | Positioning operations in the dental safety net to enhance value-based care delivery in an integrated health-care setting |  |  |  | The rate of sealant placement between 2016- 2018 revealed that FHC-M consistently exceeded rates reported statewide and nationally. For this quality indicator, performance across all dental practices in 27 states reported by Centers for Medicare and Medicaid Services in 2018 achieved 23% in 2017 compared to 73% and 52% placement rates reported by FHC-M and community health centers, respectively.  A 1:1 hygienist-to-dentist was documented across FHC-M dental centers compared to 0.5:1 reported nationally |  |  |
| Norton et al. 2022 | Medicareâ€™s Hospital Value-Based Purchasing Program Values Quality over QALYs |  |  | Incentives in HVBP value reductions in mortality at about $1.2 million per QALY, averaged across the 3 incentivized conditions. The value of a QALY in HVBP is much higher than the $50,000 to $200,000 thresholds commonly seen in medical care cost- effectiveness literature. |  |  |  |
| Noritz et al. 2017 | A Population Intervention to  Improve Outcomes in Children  With Medical Complexity |  |  | Total inpatient charges  were reduced by $11â€‰764â€‰856 | there was an 18.0% decrease in admissions and a 31.9% decrease in the average length of stay for children in the cohort. There was an 8.2% increase (P < .001) in the  percentage of children with weights between the fifth and 95th percentiles.   The care coordination program enrolled 58.3% of the cohort. |  |  |
| Nilsson et al. 2017 | Value-based healthcare as a trigger for improvement initiatives |  |  |  | Admission pathways and communication between wards were a theme as a problem to be solved by VBHC, the solution implemented was standardized care plan for patients was developed that still took the individualâ€™s needs and wishes as its point of departure. That meant that the staff worked actively and in detail on the whole care process. Care was planned in collaboration with patients at admission. This was found to create better structured care process |  |  |
| Nguyen et al. 2023 | Is value-based healthcare a strategy to achieve universal health coverage that includes oral health? An Australian case study |  |  |  |  |  |  |
| Misplon et al. 2022 | Evaluation of the implementation of Value-Based Healthcare with a weekly digital follow-up of lung cancer patients in clinical practice |  | A total of 92% of these weekly questionnaires were responded by patients. These response rates are high for patients included during a long period in the digital platform (>90%)  The majority (90%) of patients felt that this online system is easy to use |  | 95% of all lung cancer patients of the two participating thoracic oncologists agreed to participate in the digital follow-up during the period January 2018 to September 2020 (201 participating patients.  A total of 92% of these weekly questionnaires were responded by patients. These response rates are high for patients included during a long period in the digital platform (>90%)  The multidisciplinary lung cancer care team stated that the defined standard care pathways are clear for the care team and that it makes planning of care activities easier  The thoracic oncologists were the driving forces behind the installation of the multidisciplinary care team, standard care pathways, and the implementation of the digital platform. In the interviews, this was appointed as a key success factor in the implementation process. |  |  |
| Miao et al. 2019 | Constructing a value-based healthcare system for hypertensive patients through changing payment mode: evidence from a comparative study in rural China | The intervention significantly increased per capita annual outpatient expenditure by 31.8% , but decreased the per capita inpatient expenditure by â€“40.7% | At follow-up, the average DBP of the control group was 114.6 mmHg, which was significantly higher than the 109.4 mmHg of the intervention group.  There was no significant difference in SBP between both groups   The prevalence of complications increased slightly during this 1-year intervention in general, but the difference was not statistically significant | The intervention significantly increased per capita annual outpatient expenditure by 81.2 31.8%, but decreased the per capita inpatient expenditure by â€“40.7%. |  |  |  |
| Mei et al. 2015 | Value-Based Purchasing, Efficiency, and Hospital Performance |  |  | The effect of spending per patient with Medicare on clinical process of care was not significant. | Hospitals with higher spending per hospital patient with Medicare (less efficient) had lower scores for both patient experience of care and total performance, meaning lower efficiency is associated with lower quality of care.  Larger hospitals had significantly lower weighted patient experience of care scores and marginally significantly lower TPSs. However, larger hospitals had higher clinical process of care scores, although the effect is marginal.   Hospitals in a multihospital system performed significantly better in clinical process of care and total performance. However, system- owned hospitals had significantly lower patient experience of care scores. |  |  |
| McHugh et al. 2013 | An Early Look at Performance on the Emergency Care Measures Included in Medicareâ€™s Hospital Inpatient Value-Based Purchasing Program |  |  |  | Public Hospitals had the highest percentage of scores based on improvement (39.8%); for-profit hospitals had the lowest (27.8%). |  |  |
| Maki et al. 2023 | Value-based care of older peopleâ€”The impact of an acute  outreach service unit on emergency medical service  missions: A quasi-experimental study |  |  |  | There were 786 EMS missions carried out in the pre-intervention period and 654 EMS missions carried out in the post-intervention period. This is a result of decreased in non-urgent EMS missions.  only 5% of the residents treated by LiiSa  were subsequently admitted to the ED for the same symp- tom they received treatment from LiiSa |  |  |
| Makdisse et al. 2022 | Value-based healthcare in Latin  America: a survey of 70 healthcare  provider organisations from Argentina,  Brazil, Chile, Colombia and Mexico |  |  | 24.3% of organisations  informed to measure cost at the medical condition level  but only two (2.9%) measured costs of full care cycles.  In regard to cost measurement, 24.3% of organisations  informed to measure cost at the medical condition level  but only two (2.9%) measured costs of full care cycles.  Most of them measured costs only at the service or department level (67.2%), although 28.7% referred that pilots  were underway to measure costs at the condition level. Six  (8.6%) participants did not have a cost system structure in place to measure costs on a routine basis. Only one  organisation was using the TDABC methodology | The value equation was mentioned by 14 participants (24%) further only 5 of the 14 mentioned the equation as 'outcome/cost'.   Patient-reported outcome measures (PROMs) were  measured by 41.4% but in only 8.5% this information  was used to give feedback to the care team, or in routine  medical encounters with patients, or published on the  internet.  In 10 Brazilian organisations, which corresponds  to 25.6% of participants from that country, PROMs were  collected using ICHOM standard sets for heart failure,  stroke and hip and knee osteoarthritis as part of a collaborative started in 2017 by the National Association of  Private Hospitals |  |  |
| Maganty et al. 2023 |  |  |  |  |  |  | 90% of reported measures were not urology specific measure but are related to primary care |
| Maciejewski et al. 2014 | Value-Based Insurance Design Program In North Carolina Increased Medication Adherence But Was Not Cost Neutral |  | there were reductions in the probability of inpatient admissions in all three cohorts in the two years following VBID implementation. | expenditures for the three cohorts combined decreased by $5.7 million | there were reductions in the probability of inpatient admissions in all three cohorts in the two years following VBID implementation. |  |  |
| LimaRocha et al. 2022 | Efficiency in the cath lab: Pursuing value-based improvements following a sociotechnical approach |  |  | he second simulation model showed that with two Cath rooms function- ing, CRIA only needed to increase human resources capacity by 44.4% to obtain the same operational performance; at the start-up of the day even better results were obtained. Improved human resources allocation represents annual cost savings of around 136 996 euros when compared to a scenario where human resources are simply doubled. |  |  |  |
| Lichkus et al. 2019 | Effect of Implementing a Bundled-Payment Program for Heart Failure at a Safety-Net Community Hospital |  |  | Prior to bundle implementation, the average cost per episode was $29,983.04. After bundle implementation, the average cost per episode was $29,026.0  Over the 21-month study period, the hospital had a positive margin of more than $700,000 in the bundle. After splitting the savings with the convener and paying an administrative fee, LGH received more than $200,000.   The decrease in average spend on SNF and inpatient rehabilitation can be attributed to the decreased percentage of patients discharged to SNF and inpatient rehab, respectively, after bundle implementation. |  |  |  |
| Li & AL-Amin 2021 | The interaction between high-level electronic medical record adoption and hospitalist staffing levels: A focus on value-based purchasing | Initialling expensive but matures over time | Once matured, improves process and data integration | |  |  |  |
| Lee et al. 2023 | Transition to Value-based Healthcare: Development, Implementation, and Results of an Optimal Surgical Care Framework at a National Cancer Instituteâ€“designated Comprehensive Cancer Center |  |  | Reduction in costs in associated with increase resource availability, with a 134% increase in surgical volume.  13% reduction in total OR costs after adjusting for inflation | Over 5 year, Optimal Surgical Care rates demonstrated improvement across all procedure buckets, accompanied by a 134% increase in surgical volume |  |  |
| Lawrence et al. 2020 | Variability in skilled nursing facility screening and admission processes: Implications for value-based purchasing |  | inconsistent and inadequate transfer of medical documentation,  Payment models that encourage hospitals to discharge patients quicker and sicker. |  | Lack of understanding among hospital staff of SNF protocols and capabilities. Use of different approaches to screen patients for admission to SNF, verse types of gatekeepers to manage patient admission to SNF Different approaches to monitoring and managing patients once admitted. |  |  |
| Lassen et al. 2020 | Do Bundled Payment Programs in Joint Replacement Care Hold Promise for Improving Patient Outcomes? |  | Patients at BPCI hospitals had significantly higher satisfaction scores than patients at CJR hospitals | current findings do not show that BPCI hospitals have higher net patient revenue per bed after academic affiliation, geographic region, and efficiency score are controlled. |  |  |  |
| LaFave et al. 2021 | The Value of Home-Based Primary Care:  Qualitative Exploration of Homebound  Participant Perspective |  | HBPC provides peace of mind by ensuring access to care for older patients, as well as supports patients living in home | |  |  |  |
| Labovitz et al. 2017 | Web-Based Patient Experience Surveys to Enhance Response Rates |  |  | Web-based survey administration costs two to four times less than standard mail, phone, and mixed-modal survey administration | 87 completed surveys (65.9%), with no significant differences among distribution methods |  |  |
| Kumar et al. 2022 | Impact of Hospital-Based Rehabilitation Services on Discharge to the Community by Value-Based Payment Programs After Joint Replacement Surgery |  |  |  | a hospitalâ€™s participation in value-based payment programs was not significantly associated with community discharge for patients after THR and TKR  significant positive association between a higher amount of rehabilitation services during acute hospitalization with a greater likelihood of community discharge in patients after hip and knee joint replacement |  |  |
| Kukreja et al. 2021 | Utilizing time-driven activity-based costing to determine open radical cystectomy and ileal conduit surgical episode cost drivers |  |  | Total inpatient care was the main driver of cost for radical cystectomy care making up 32% of the total costs, mainly being patient staff care at 76%  Consumables accounted for 17% of costs  Surgery contributed 31% of the costs, with the majority derived from operating room staff costs at 71% | Mean length of stay 6.9 days |  |  |
| Koy et al. 2023 | The Flipped Break-Even: Re-Balancing Demand- and Supply-Side Financing of Health Centers in Cambodia |  |  | 93.94% of total funding comes from the government as supply-side funding, and 51.83% of the total income is independent of the performance of the facility,  32.71% of the total income is provided by the government in the form of essential drugs, medical materials, and vaccines. |  |  |  |
| Koster et al. 2023 | Dealing with Time Estimates in Hospital Cost Accounting: Integrating Fuzzy Logic into Timeâ€‘Driven Activityâ€‘Based Costing |  |  | The total cost for treating a RA patient during the  first year following diagnosis is calculated at â‚¬1497 using  the FL-TDABC methodology.  The diagnostics phase rep- resents 32% of the total costs   The one-sample t test showed a significant difference in costs between the FL-TDABC and ABC (DTC) methodology at a 5% alpha level (p = 0.0101, t = 2.566) |  |  |  |
| Koressel et al. 2022 | Profound Impact of Insurance Payor and Socioeconomic Status in Total Hip Arthroplasty Outcomes: Results From a High Volume Tertiary Care Center |  | dual eligible patients: - stayed in the hospital over half a day longer - less likely to be discharged home - more likely to present back to the emergency department (ED) within 90 days after their procedure |  |  |  |  |
| Koolmees et al. 2022 | Time-Driven Activity-Based Costing Accurately Determines Bundle Cost for Rotator Cuff Repair |  |  | the bundled cost of care for RCR is $10,569 using TDABC method.  cost drivers were orthopedic surgeon, suture anchors, and physical therapy-related cos |  |  |  |
| King et al. 2021 | Prioritization framework for improving the value of care for very low birth weight and very preterm infants |  |  | the top 10 high priority CTT categories were responsible for $185,820,182 in costs (66% of all costs from included CTT categories), and include many commonly used tests and treatments in neonatal care |  |  |  |
| Karim et al. 2021 | Financial Performance of Hospitals in the Appalachian Region Under the Hospital Readmissions Reduction Program and Hospital Value-Based Purchasing Program |  |  | only hospitals in Appalachian states experienced a significant reduction in operating margin. |  |  |  |
| Jones et al. 2019 | Observations on the Medicare Value- Based Ranking of Hospitals During  Fiscal Years 2015 and 2016 |  |  |  | Higher performing hospitals contribute less to education, research and indigent care |  |  |
| Jayakumar et al. 2023 | A Model for Evaluating Total Costs of Care and Cost Savings of Specialty Condition-Based Care for Hip and Knee Osteoarthritis in an Integrated Practice Uni |  |  | Weighted average costs of IPU-based nonoperative management for hip OA ($475) were lower than traditional nonoperative management ($795) and lower in IPU-based operative management including a total hip replacement (THR) pathway ($15 902) than traditional operative management ($16 162)  The weighted average costs of IPU-based nonoperative management for knee OA ($464) were lower than traditional nonoperative management ($854) and lower in IPU-based operative management including a total knee replacement (TKR) pathway ($15 738) than traditional operative management ($15 918)  IPU-based outpatient care saves 40.3% ([$795-$475)/$795]) per patient with hip OA and 45.7% ([$854-$464)/$854]) per patient with knee OA. |  |  |  |
| Jain et al. 2019 | Strategies for Delivering Value-Based Care:  Do Care Management Practices Improve  Hospital Performance? |  |  | Care Management Processes increase costs per Medicare beneficiary | Not a significant change in performance |  |  |
| Ingraham et al. 2016 | Reductions in High-End Imaging Utilization With Radiology Review and Consultation |  |  | Results demonstrate that radiologists' review and consultation reduced the quantity of high- end outpatient imaging performed and improved the quality and appropriateness of imaging selected |  |  |  |
| Hoong et al. 2023 | Impact of the value driven outcomes  program among cataract surgery patients  in Singapore: an interrupted time series analysis |  | patients after the implementation of the VDO program tended to have slightly better outcomes. | after the implementation of the VDO pro- gram, the mean total costs had a statistically significant  level decrease by $283.76 | the total cost of  cataract surgery significantly decreased in trend over  time, while quality outcomes remained high. |  |  |
| Hennick et al. 2013 | Value-based healthcare in Lynch syndrome |  | 5 outcomes identified by patients as being important | | 6 outcomes identified as being relevant to patients |  |  |
| Harris et al. 2019 | Examining and Understanding Value: The Cost of Preoperative Characteristics, Intraoperative Variables and Postoperative Complications of Minimally Invasive Partial Nephrectomy |  |  | BMI did not seem to significantly impact cost when examined in a binary fashion or when stratified into multiple variables  Patients 56 to 65 years old had a significantly higher Direct Costs at$12,500  Patients with Diabetes had an increased median direct costs of $12,300  Nephrometry score was not associated with a difference in cost parameters  patients who underwent unplanned reintubation was $26,000  Patients who had a blood transfusion and median direct costs was $15,200 |  |  |  |
| Harold et al. 2019 | Single-Use Custom Instrumentation in Total  Knee Arthroplasty: Effect on In-Hospital  Complications, Length of Stay, and Discharge  Disposition |  |  |  | CI TKA did not reduce operative time, transfusion rate,  postoperative haemoglobin drop, haemovac  output, or hospital length of stay |  |  |
| Hale et al. 2021 | Improving Medication Adherence in an ACO Primary Care Office With a PharmacistLed Clinic: A Report From the ACORN SEED |  |  | Increased adherence to medication translated to notable cost savings |  |  |  |
| Gronbeck & Feng 2023 | Performance and Quality Measure Selection by Mohs Surgeons in the 2020 Merit-Based Incentive Payment System |  |  |  |  | Mohs that exceeded the performance threshold received a merit-incentivised payment | 77.4% exceeded performance threshold |
| Grabowski et al. | The Impact of Nursing Home Pay-for- Performance on Quality and Medicare Spending: Results from the Nursing Home Value-Based Purchasing Demonstration |  |  | No change in costs | No improvement in quality of care |  |  |
| Goretti et al. 2020 | Value-Based Healthcare and Enhanced Recovery After Surgery Implementation in a High-Volume Bariatric Center in Italy |  | Percentage Excess Weight Loss was 82.03%, steadily higher than the European average (51.89%) 3 years after surgery.  Patients recovered from comorbidities, thus, discontinuing or reducing pharmacological treatment.   Seventy-seven percent of the patients reported to work better and more than before the procedure.  patients spent 40% less time at the hospital completing all the exams in a single morning | Decrease in usage of: Chest X-ray Postoperative intensive care unit usage | Both readmission and reoperation rates were 0.4% within 30 days of surgery.  Patients spent 40% less time at the hospital completing all the exams in a single morning  Because of the new anesthetic protocol and the early post-surgical mobilization (30 min after surgery), patients had better pain and vomiting control, leading in turn to 40% lower drug prescriptions |  |  |
| Glasgow et al. 2019 | Hospital palliative care consult improves value- based purchasing outcomes in a propensity  scoreâ€“matched cohort |  |  | $2350 per day in savings when patients are seen by palliative care. | Reduced hospital admissions |  |  |
| Ghisleni et al. 2023 | Value-based health care in heart failure: Quality of life and cost analysis |  | More than 30% of the sample was characterized by moderate-severe anxiety/depression symptoms | Based on the New York Heart Association classifications:  The medication total cost was similar between NYHA I and NYHA III/IV(US$ 123.94 vs. US$ 135.08), but it corresponded to 57% of the total treatment cost in NYHA I patients, while in NYHA III/IV, it corresponded to 20%.   A different pattern is evident in lab/exam costs, which represented almost 30% of the costs in NYHA I, and 74% in NYHA III/IV (US$ 63.26 vs. US$ 491.05). |  |  |  |
| Ganske et al 2021 | Time-Driven, Activity-Based Costing of  Presurgical Infant Orthopedics: A Critical  Component of Establishing Value of Latham  Appliance and Nasoalveolar Molding | Nasoalveolar molding requires more frequent clinic visits |  | Both treatments can be completed for low cost |  |  |  |
| Galvez et al. 2020 | Value-Based Healthcare in Ostomies |  | We found significant and (statistically) relevant differences from a clinical perspective both in gained utility (QALYs) and in quality of life measured with the specific questionnaire for ostomy patients. | Patients showed a significant increase in their demand for consultations with specialized nurses and a decrease in demand for other more congested and specialized services, such as emergency hospital treatment, primary care, or hospital specialists. This supposes an important saving to the health system, in terms of both cost and time.   The implementation of the new management model for patients with ostomies in our region offers a very efficient alternative: â‚¬2297/QALY. This is especially true if we consider that, in Spain, a technology, a new treatment or procedure, is considered to be efficient when the cost of gaining 1 QALY ranges between â‚¬22,000 and â‚¬24,000 | We found significant and (statistically) relevant differences from a clinical perspective both in gained utility (QALYs) and in quality of life measured with the specific questionnaire for ostomy patients.  The percentage of patients with complications arising from the stoma (28.8%) suggests good management of surgical techniques and good health education in self-care. This is due to nurses specializing in ostomy care dedicated to training in self-care |  |  |
| Gabriel et al. 2019 | Value-based healthcare analysis of joint  replacement surgery for patients with  primary hip osteoarthritis |  |  | Both models delivered good clinical outcomes. However,  model 2 delivered better value given that the associated  costs were lower. | There was no significant difference in PROMs as assessed by either the traditional or reformatted outcome assessment techniques.  The utilisation of Integrate Practice Unit (IPU) model allowed for a single point of entry rather than multiple leading to less wait times, leading to surgery faster |  |  |
| Gabe-Walters et al. 2021 | Lymphoedema care in the mobile unit: a service evaluation of patient experience  and attendance rates |  | Increased access to care is noted.  98% of respondents cited high levels of satisfaction  with the service. |  | Patients provide positive feedback regarding the staff of the mobile cancer unit   Overall preference for attending the mobile unit instead of a hospital-based service was expressed by 89% of respondents, while 9% felt that the two experiences were comparable. |  |  |
| Fortmann et al. 2020 | Care Team Integration in Primary Care Improves One-Year Clinical and Financial Outcomes in Diabetes: A Case for Value-Based Care |  | CMC-TI participants achieved statistically significant improvements in healthful eating (4.20 to 4.96 days/week), exercise , blood glucose monitoring, and foot-checking ; no significant change was observed for self-reported medica- tion adherence. Small, but statistically significant decreases were observed for diabetes distress over 1 year; the percentage of individuals reporting â€˜â€˜moderateâ€™â€™ distress decreased from 24.1% to 14.3% during this period. | The CMC-TI group exhibited significantly greater reductions on all inpatient utilization and cost outcomes relative to usual care | The CMC-TI group exhibited significantly greater improvements in HbA1c over 1 year compared with usual care |  |  |
| Fong et al. 2022 | Medicaid long-term care workforce training intervention and value-based payment metrics |  | Clients less likely to experience uncontrolled pain. | | Increased flu vaccination occurred at a population level amongst clients. |  |  |
| Fidanza et al. 2022 | What Is the Inpatient Cost of Hip Replacement? A Time-Driven Activity Based Costing Pilot Study in an Italian Public Hospital |  |  | The total cost of total hip arthroplasty is EUR 6002.06  The most common direct cost is the purchase price of the implant  Reducing length of stay is a process improvement that will reduce costs |  |  |  |
| Feizpour et al. 2021 | Enhanced recovery in liver transplantation: A value-based approach to complex surgical care |  | ERP improves recipient outcomes with reduce lengths of stay and admissions to ICU | improvement in outcomes led to median cost reductions of $11,409 and $29,409 per patient compared to pre-ERP recipients and intubated post-ERP recipients, respectively. | Decreased operating time and optimal resource allocation occurred |  |  |
| Farley et al. 2023 | Implementation outcomes associated with a value-based care model of comprehensive medication management in community pharmacies |  | Increased pharmacist engagement | Incentives for achieving clinical and patient outcomes |  |  |  |
| Etges et al. 2022 | Telemedicine Versus Face-to-Face Care in Ophthalmology: Costs and Utility Measures in a Real-World Setting |  | no significant difference (for most do- mains) in patient-perceived quality of life as measured by the utility of a telediagnosis service in ophthalmology when compared with face-to-face care in the same specialty. | 31% cost saving providing telehealth compare to face to face care |  |  |  |
| Etges et al. 2022 | Moving the Brazilian ischaemic stroke pathway to a  value-based care: introduction of a risk-adjusted cost  estimate model for stroke treatment |  | High risk patients tend to have a greater Length of Stay  Patients with favourable outcomes, mRS (0â€“2) and  treated with any therapy demonstrated a similar cost composition and LOS distribution between the phases of the care  pathway | Patients with a higher modified Rankin Score (mRS) used more resources therefore costed more. High risk patients tend to have a greater Length of Stay |  |  |  |
| Ennis et al. 2019 |  |  |  | model is useful because it aggregates patients in similar clinical situations to analyze their cost of care.  hospital admissions and ED visits are important drivers of cost in castration-resistant disease | facilitates the comparison of patients with similar disease states but with different treatment approaches |  |  |
| Ellis et al. 2017 | Refining Our Understanding of  Value-Based Insurance Design and  High Cost Sharing on Children | The results showed that differences  across plans in OOP costs were  sizeable and that differences in total  spending across health plans which  differ dramatically in their OOP cost  burdens were modest.  total spending  per child broadly increased across  family income, ranging from $10â€‰000  to $50â€‰000, before remaining nearly  constant at higher income levels. |  |  |  |  |  |
| Dolce et al. 2020 | Interprofessional value-based health care: Nurse practitioner-dentist model |  | systolic BP decreased postintervention as well as weight, BMI, and HbA1c.   Positive increase in the proportion of patients who had advanced care planning post- intervention |  |  |  |  |
| Dohmen et al. 2022 | Implementing value-based healthcare using a digital health exchange platform to improve pregnancy and childbirth outcomes in urban and rural Kenya |  |  | Further investment is needed to train providers on how to manage financial risks related to bundled payment |  |  |  |
| Depla et al. 2022 | Patient-Reported Outcome and Experience Measures in Perinatal Care to Guide Clinical Practice: Prospective Observational Study |  | In several domains, such as incontinence and breastfeeding, the high alert rates revealed opportunities to improve and personalize perinatal care for individual women on outcomes that matter to them.  The mental health domain indicate that the first instrument of the 2-step screening is missing an unacceptable proportion of women at risk for depression |  | some measurement instruments and their timing as proposed by ICHOM are less suitable for clinical use. |  |  |
| Demedts et al. 2021 | Clinical implementation of value based healthcare: Impact on outcomes for  lung cancer patients |  | VBHC for lung cancer  patients is feasible in routine clinical practice and that this is beneficial for the individual lung cancer patient, resulting in less emergency department visits, shorter length of stay on the oncology day clinic and  better survival. | Less emergency department visits, shorter length of stay on the oncology day clinic can decrease costs per patient |  |  |  |
| Dekkers et al. 2022 | Costs and Its Determinants in Pituitary Tumour Surgery |  |  | Length of hospital stay, and complications are the main drivers of costs in perioperative pituitary tumour healthcare. Mean cost per patient = 16,339 euro |  |  |  |
| de Jong et al. 2020 | Cost-effectiveness of Telemedicine-directed Specialized vs Standard Care for Patients With Inflammatory Bowel Diseases in a Randomized Trial |  | Increase in QALYs | Telemedicine found to be more cost-effective | Early identification of patients with insufficiently controlled disease |  |  |
| Daruwalla et al. 2023 | INTRAVITREAL ANTI-VASCULAR ENDOTHELIAL GROWTH FACTOR PHARMACEUTICAL UTILIZATION AND FINANCIAL IMPACT IN A VALUE-BASED CARE PROGRAM |  | The COVID-19 Pandemic reduced patient appointments due to risk of exposure, however the decrease in appropriate use of anti-VEGF can lead to negative ophthalmic health outcomes, such as decreased best-corrected visual acuity and worsened anatomic results |  |  |  |  |
| Daras et al. | Nearly One In Five Skilled Nursing Facilities Awarded Positive Incentives Under Value-Based Purchasing |  |  | a substantial percentage of SNFs (26 percent in FY 2019 and 19 percent in FY 2020) were able to earn incentive payments in excess of what they would have received without the program.  the majority of SNFs earned less in Medicare reimbursement as a result of their performance in the program, which is statutorily required to ensure Medicare savings. | SNFs that were not for profit, government owned, located in rural areas, and larger were significantly more likely to earn positive incentive payments after selected facility characteristics and geographic region were controlled for |  |  |
| Daniels et al. 2022 | Five yearsâ€™ experience with value-based  quality improvement teams: the key factors  to a successful implementation in hospital care |  |  |  |  |  |  |
| daSilvaEtges et al. 2022 | Time-driven activity-based costing as a strategy to  increase efficiency: An analyses of interventional  coronary procedures |  |  | the mean cost per patient varied across hospitals from $807 to $2639   The mean cost difference between the  highest and lowest cost per patient at the post-procedure phase was $384 compared to the procedure phase (mean cost difference being $71) | higher costs in the post-procedure phase were due to hospital structure use in this phase, as its cost is directly  proportional to patient's length of stay. |  |  |
| Cwalina et al. 2022 | Group practice in Urology:  A cross-sectional analysis  over 8years (2014â€“2021) |  |  |  | Adjustments in reimbursement models  have re-centered practice operations towards quality and  cost reduction. Large medical practices are likely best  equipped to meet these demands through access to favour- able capital investment and technology. |  |  |
| Cramer-VanDerWelle et al. 2021 | â€˜Care for Outcomesâ€™: systematic  development of a set of outcome  indicators to improve patient-relevant  outcomes for patients with lung cancer |  |  | A smaller number of indicators reduces administrative burden | Introduction of outcome measures can be introduced and data retrospectively collected. |  |  |
| Cohen et al. 2021 | Surgical Management of Obesity in Brazil: Proposal for a Value-Based Healthcare Model and Preliminary Results |  |  | 7.1% reduction in costs with the VBHC model | Less complications, less ICY admissions, less readmissions in the VBHC model |  |  |
| Chiu et al. 2022 | Association of the Medicare Value-Based Purchasing Program With Changes in Patient Care Experience at Safety-net vs Nonâ€“Safety-net Hospitals |  |  |  | patient-reported experience across measures of global satisfaction, communication, processes of care, and hospital environment did not differentially improve at safety-net hospitals compared with nonâ€“safety-net hospitals in the 8 years after the implementation of the VBP program |  |  |
| Chen et al. 2017 | Financial Performance of Hospitals in the Mississippi Delta Region Under the Hospital Readmissions Reduction Program and Hospital Value-based Purchasing Program |  |  | Post implementation, Delta hospital operating margin decreased by 0.89% |  |  |  |
| Chatterjee et al. | Patient Experience in Safety-Net Hospitals |  |  |  | Safety Net Hospitals perform more poorly than other hospitals n nearly every measure of patient experience and that gaps in performance were size able and persistent over time |  |  |
| Chatfield et al. 2019 | Bending the cost curve: time series  analysis of a value transformation  programme at an academic  medical centre |  |  | This initiative was associated with a 7.7%  drop in mean monthly variable direct cost per case,  cutting institutional expenses by $59.3 million to  date | While making cost savings, no negative impacts on 30-day same-hospital readmissions or in-hospital mortality. |  |  |
| Chatfield 2016 | Value-Based Purchasing |  |  | The data clearly show that for-profit hospitals and small hospitals ( 99 beds) are out-performing hospitals that are larger and thatare operating under nonprofit or government ownership structures in regard to VPB PC measures, HCAHPS measures, and TPS | The data clearly show that for-profit hospitals and small hospitals ( 99 beds) are out-performing hospitals that are larger and thatare operating under nonprofit or government ownership structures in regard to VPB PC measures, HCAHPS measures, and TPS |  |  |
| Calderon et al. | Questionable validity of the catheter-associated urinary tract infection metric used for value-based purchasing |  | CDC NHSN CAUTI metric used by the Centers of Medicare and Medicaid Services may be invalid due to the large statistical difference between this metric and the AHRQ CAUTI metric. The difference being that the latter suggests there's a 28% decrease in performance improvement for catheter-associated urinary tract infections | | |  |  |
| Burke et al. 2022 | Skilled Nursing Facility Performance and Readmission Rates Under Value-Based Purchasing |  |  | program as currently structured rewards SNFs that are already high performing and penalizes those with low baseline performance, even if these SNFs achieved the top levels of improvement. |  |  |  |
| Bueno et al. 2022 | The Clinical outcomes, healthcare resource utilization, and related costs (COHERENT) model. Application in heart failure patients |  | The COHERENT model allows the inclusion of several of the most relevant outcome measures for assessing treatment results and can be used for outcome comparison between patient subgroup | 144.91 euro per patient/day.  model allows measurement and calculation of costs for observation stays, an increasingly important health care resource use, with heterogeneous pathways and billing models, often ignored in readmission measures and quality indices. |  |  |  |
| Borden & Blustein 2012 | Valuing Improvement in Value-Based Purchasing | A more equitable definition of improvement allocates more funding to hospitals in income poor areas | The change in definition of improvement incentives low performing hospitals more than high performing hospitals. | A change in the definition of improvement will reward hospitals in low socio-economic areas | More fairness in improvement scores will incentivise improves in quality |  |  |
| Bodar et al. 2020 | Time-Driven activity-based costing identifies opportunities for process efficiency and cost optimization for robot-assisted laparoscopic pyeloplasty |  |  | Robot-assisted Laparoscopic Pyeloplasty costs is 18% lower than traditional surgery |  |  |  |
| Bigger et al. 2021 | Advance Care Planning Protocols and Hospitalization Rates in Home Health Value-Based Purchasing |  | The greater an agencyâ€™s ACPP score, the higher its hospitalization rates | | The greater an agencyâ€™s advance care planning protocols score, the higher its hospitalization rates |  |  |
| Bensink et al. 2023 | Value-based healthcare in fertility care  using relevant outcome measures for the  full cycle of care leading towards shared  decision-making: a retrospective  cohort study |  | Increased transparent dialogue between provider and patient | |  |  |  |
| Basto et al. 2019 | Time-driven activity-based costing to model the utility of parallel induction redesign in high-turnover operating lists |  |  | With an increase in intraoperative personnel costs under a parallel induction design of $50 with no additional case and $397 with an additional case, the prospect of further case throughput represents substantial value improvement given average single day admission revenue of $2818 per short case | median 11-min reduction in non-operative time |  |  |
| Banerjee et al. 2019 | Association between degree of exposure to the Hospital Value Based Purchasing Program and 30-day mortality: experience from the first four years of Medicareâ€™s pay- for-performance program |  | no significant impact of pay-for-performance programs on patient outcomes |  | no significant impact of pay-for-performance programs on patient outcomes |  |  |
| Ayoube et al. 2023 | Redefining â€œValueâ€ in Surgery: Development of a Comprehensive Value Score for Outpatient Endocrine Surgery | Demonstrated the cost impact telehealth vs in person appointments | Efficacy of Intervention not assessed fully | Framework allows for assessment of intervention | Framework allows for assessment of intervention |  |  |
| Artenstein et al. 2021 | An Integrated Mobile Acute Care Service Enhances Value |  | Patient satisfaction with service | decreased costs through decreased hospital presentations estimate at $1729 USD per visit | Reduces unnecessary utilisation of resources |  |  |
| Arenchild et al. 2020 | Do We Get What We Pay For?  Examining the Relationship Between  Payments and Clinical Outcomes in  High-Volume Elective Surgery in a  Commercially-Insured Population |  |  |  | No relationship between healthcare costs and quality. |  |  |
| Alibrahim Et al. 2021 | Towards value-based healthcare: Establishing  baseline pharmacy care costs for diabetes  management |  |  | Effective process improve will reduce costs |  |  |  |
| Alibrahim et al. 2022 | Towards value-based healthcare: Establishing  baseline pharmacy care costs for diabetes  management |  |  | estimates can enable value-based care in three ways:  (1) value-based cost-effectiveness analyses of care redesigns and interventions,  (2) comparative cost assessments as a form of benchmarking, and  (3) value-based healthcare payment arrangements |  |  |  |
| Al-Amin et al. 2023 | Were hospitals with sustained high performance more successful at reducing mortality during the pandemicâ€™s second wave? |  |  |  | Sustainers had lower mortality rates than nonsustainers during the second wave of COVID-19 |  |  |
| Ahn et al. 2019 | Defining and Optimizing Value in Total Joint Arthroplasty From the Patient, Payer, and Provider Perspectives |  | Diabetes effected the ability of patients to recover post surgery. |  | Low value total joint arthroplasty for patients returning to skilled nursing facilities, and patients with pulmonary disease |  |  |
| Ahluwalia 2021 | Improving the efficiency of ankle fracture care through home care and day-surgery units: Delivering safe surgery on a value-based healthcare model |  | Increased patient satisfaction with the service, | Proposed intervention more efficient reducing costs in a Time Driven Activity Based Costing model | Proposed intervention clinically safe, decreased length of stay |  |  |
| Adler-Milstein 2022 | Association of Primary Care Engagement in Value-Based Reform Programs With Health Services Outcomes |  |  | Participation in MU-MSSP jointly resulted in savings of 0.51% and MSSP alone of 0.55% | Improved outcomes are noted with participation in one program while findings were inconsistent with participation in all 3 reform programs |  |  |
| Abbott 2011 | A Microcosting Approach for Isolated, Unilateral Cleft Lip Care in the First Year of Life |  |  | The overall median cost was $13,013. The operation represents 68% of costs while inpatient ward care is 19%. There is a potential to reduce costs by more than 10% if the intervention was completed as an outpatient procedure. |  |  |  |

**Appendix F:** Models of Care and Funding

| Study ID | Title | Models of care | Funding model |
| --- | --- | --- | --- |
| Zhu et al. 2019 | The Cost to Attending Surgeons of Resident Involvement in Academic Hand Surgery |  |  |
| Zhao et al. 2022 | Telehealth and hospital performance: Does it matter? | tele/videoconference | Value Based Purchasing |
| Zhang & Cowling 2023 | Association of Participation in a Value-Based Insurance Design Program With Health Care Spending and Utilization | Outpatient | Other |
| Yuhua et al. 2017 | Value-based payment in implementing evidence-based care: the  Mental Health Integration Program in Washington State | Outpatient | Value Based Purchasing |
| Yu et al. 2017 | Time-driven activity-based costing: A dynamic value assessment model in pediatric appendicitis | In hospital | Time-Driven Activity-Based Costing |
| Wong 2023 | Dialysis Costs for a Health System Participating in Value-Based Care | In hospital ; Outpatient | Value Based Purchasing |
| Wolosin et al. 2012 | Nursing Care, Inpatient Satisfaction, and Value-Based Purchasing |  |  |
| Wohlin et al. 2021 | As predicted by theory: choice and competition in a publicly funded and regulated regional health system yield improved access and cost control | In hospital | Bundle Car Payment |
| Wittlieb-Weber et al. 2015 | Pediatric Versus Adult Cardiomyopathy and Heart Failure-Related Hospitalizations: A Value-Based Analysis | In hospital |  |
| Wickman et al. 2022 | Influence of medical comorbidity and surgical indication on total elbow arthroplasty cost of care |  |  |
| Weiss et al. 2019 | Effective Care Management for  Children With Special Health  Care Needs in the Era of  Value-Based Payment | Outpatient | Capitation |
| VanHooff et al. 2017 | Care for Outcomesâ€™: systematic  development of a set of outcome  indicators to improve patient-relevant  outcomes for patients with lung cancer |  |  |
| van Egdom et al. 2019 | Implementation of Value Based Breast Cancer Care | Outpatient |  |
| vanDeena et al. 2017 | The impact of value-based healthcare for inflammatory bowel diseases on healthcare utilization: a pilot study | In hospital |  |
| Trenaman et al. 2023 | Medicare Beneficiariesâ€™ Perspectives on the Quality of Hospital Care and Their Implications for Value-Based Payment | In hospital | Value Based Purchasing |
| Tragl et al. 2023 | Who counts when health counts? A case-study of multi-stakeholder initiative to promote value-creation in Swedish healthcare |  |  |
| Tozzi et al. 2023 | Using big data and Population Health  Management to assess care and costs  for patients with severe mental disorders  and move toward a value-based payment  system |  |  |
| Thomas et al. 2021 | Changing the process of prescribing to  procuring lymphoedema compression  garments: a service evaluation | Outpatient | Fee for Service |
| Teshale et al. 2021 | Early Effects of Home Health Value-Based  Purchasing on Quality Star Ratings | Outpatient | Value Based Purchasing |
| Tavengwa et al. 2023 | A pilot study to explore societal, patient, and public authority perception on â€˜Value- Added Taxâ€™ system for healthcare financing in Zimbabwe: A case for cancer treatment |  |  |
| Tanet al. 2016 | Using Quality Improvement Methods and Time-Driven Activity- Based Costing to Improve Value- Based Cancer Care Delivery at a Cancer Genetics Clinic | Outpatient | Time-Driven Activity-Based Costing |
| Stone et al. 2022 | Understanding the Economic Impact of an Essential  Service: Applying Time-Driven Activity-Based Costing  to the Hospital Airway Response Team | In hospital | Time-Driven Activity-Based Costing |
| Stanberry et al. 2021 | Using the MEAT VBP Framework to analyse and understand the value of surgical gloves: an explanatory case study |  |  |
| Sreeram et al. 2021 | Patient-Reported Outcome Measures and Clinical Outcomes in Children with Foregut Anomalies | In hospital |  |
| Spaulding et al. 2021 | Race to the Top of the Hospital Value-Based  Purchasing Program |  | Pay for Performance; Value Based Purchasing |
| Spaulding et al. 2020 | Do MagnetÂ®-Designated Hospitals Perform Better on Medicare's Value-Based Purchasing Program? |  | Pay for Performance; Value Based Purchasing |
| Spaulding et al. 2018 | Hospital Value-Based Purchasing Performance: Do Organizational and Market Characteristics Matter? | In hospital | Value Based Purchasing |
| Snow 2023 | Managing Total Knee Replacement Under Value- Based Payments | Outpatient | Fee for Service |
| Sethi et al. 2021 | Utilizing Lean Methodology and Time-Driven Activity-Based Costing Together | In hospital | Time-Driven Activity-Based Costing |
| Sethi et al. 2022 | Combining time-driven activity-based costing and lean  methodology: an initial study of single-level lumbar fusion  surgery to assess value-based healthcare in patients  undergoing spine surgery | In hospital | Time-Driven Activity-Based Costing |
| Schoonbeek et al 2021 | Determinants of delay in the head and neck oncology care  pathway: The next step in value-based health care |  |  |
| Sanchez-Gavilan et al. 2022 | Added value of patientâ€‘reported outcome  measures (PROMs) after an acute stroke  and early predictors of 90 days PROMs | In hospital |  |
| Salampessy et al. 2018 | The effect of cost-sharing design characteristics on use of health care recommended by the treating physician; a discrete choice experiment | Outpatient | Other |
| Ryan et al. 2017 | Changes in Hospital Quality Associated  with Hospital Value-Based Purchasing | In hospital | Value Based Purchasing |
| Russell et al.2020 | Longitudinal Trends in Costs for Hospitalizations at Childrenâ€™s Hospitals | In hospital |  |
| Rosenthal et al. 2007 | Employersâ€™ Use of Value-Based Purchasing Strategies |  | Value Based Purchasing |
| Rocque et al. 2017 | Resource Use and Medicare Costs During Lay Navigation for Geriatric Patients With Cancer | Outpatient | Fee for Service |
| Robards et al. 2022 | A value-based approach to prostate cancer image-guidance in a regional  radiation therapy centre: a cost-minimisation analysis | In hospital | Other |
| Riley et al. 2019 | A framework for oral health care value-based payment approaches | Outpatient ; Other | Fee for Service ; Capitation; Pay for Performance |
| Reilly et al. 2020 | Creating a Value Dashboard for Orthopaedic Surgical Procedures | In hospital | Pay for Performance |
| Reif et al. 2021 | Effectiveness of value-based purchasing for substance use treatment engagement and retention | Outpatient | Pay for Performance |
| Rangnekar et al. 2015 | The Relationship Between Hospital  Value-Based Purchasing Program  Scores and Hospital Bond Ratings | In hospital |  |
| Ramirez & Brennan 2020 | Using the value-based care paradigm to compare physical therapy access to care models in cervical spine radiculopathy: a case report | Outpatient |  |
| Ramirez et al. 2016 | Physician-Owned Surgical Hospitals Outperform Other Hospitals in Medicare Value-Based Purchasing Program | In hospital | Pay for Performance; Value Based Purchasing |
| Qi 2020 | Performance and Penalties in Year 1 of the Skilled Nursing Facility Value-Based Purchasing Program | Outpatient | Value Based Purchasing |
| Pestka et al. 2020 | Community pharmacists' perceptions  of acceptability, appropriateness, and  feasibility of a value-based care model for  comprehensive medication management | Outpatient ; tele/videoconference | Fee for Service ; Pay for Performance |
| Perera et al. 2022 | The Effects of Home Health Value-Based  Purchasing on Home Health Care Quality  in For-Profit and Nonprofit Agencies: A  Comparative Interrupted Time-Series  Analysis, 2012â€“2018 | Outpatient | Value Based Purchasing |
| Parra et al. 2017 | Assessing value-based health care delivery for haemodialysis | In hospital |  |
| Panchal et al. 2023 | The implementation of value-based frameworks,  clinical care pathways, and alternative payment  models for cancer care in the United States |  | Other |
| Orlandi et al. 2023 | Multi-level analysis and evaluation of organizational improvements  in thoracic surgery according to a Value-Based HealthCare  approach | In hospital |  |
| Okeke et al. 2021 | Implementing Value-Based Primary Care Delivery in Federally Qualified Health Centers |  | Value Based Purchasing |
| O'Donnell et al. 2023 | Coaching to Bedside Shift Report and Its Correlation to Hospital Consumer Assessment of Healthcare Providers and Systems and Value-Based Purchasing Dimension Scores | In hospital | Value Based Purchasing |
| Nycz et al. 2020 | Positioning operations in the dental safety net to enhance value-based care delivery in an integrated health-care setting | Outpatient |  |
| Norton et al. 2022 | Medicareâ€™s Hospital Value-Based Purchasing Program Values Quality over QALYs | In hospital | Value Based Purchasing |
| Noritz et al. 2017 | A Population Intervention to  Improve Outcomes in Children  With Medical Complexity | In hospital ; Outpatient |  |
| Nilsson et al. 2017 | Value-based healthcare as a trigger for improvement initiatives | In hospital |  |
| Nguyen et al. 2023 | Is value-based healthcare a strategy to achieve universal health coverage that includes oral health? An Australian case study | Outpatient |  |
| Misplon et al. 2022 | Evaluation of the implementation of Value-Based Healthcare with a weekly digital follow-up of lung cancer patients in clinical practice | In hospital ; tele/videoconference |  |
| Miao et al. 2019 | Constructing a value-based healthcare system for hypertensive patients through changing payment mode: evidence from a comparative study in rural China | In hospital ; Outpatient | Fee for Service ; Other |
| Mei et al. 2015 | Value-Based Purchasing, Efficiency, and Hospital Performance | In hospital | Value Based Purchasing |
| McHugh et al. 2013 | An Early Look at Performance on the Emergency Care Measures Included in Medicareâ€™s Hospital Inpatient Value-Based Purchasing Program | In hospital | Value Based Purchasing |
| Maki et al. 2023 | Value-based care of older peopleâ€”The impact of an acute  outreach service unit on emergency medical service  missions: A quasi-experimental study | Outpatient ; hospital in the home |  |
| Makdisse et al. 2022 | Value-based healthcare in Latin  America: a survey of 70 healthcare  provider organisations from Argentina,  Brazil, Chile, Colombia and Mexico |  |  |
| Maganty et al. 2023 |  | In hospital ; Outpatient | Value Based Purchasing |
| Maciejewski et al. 2014 | Value-Based Insurance Design Program In North Carolina Increased Medication Adherence But Was Not Cost Neutral | Outpatient |  |
| LimaRocha et al. 2022 | Efficiency in the cath lab: Pursuing value-based improvements following a sociotechnical approach | In hospital |  |
| Lichkus et al. 2019 | Effect of Implementing a Bundled-Payment Program for Heart Failure at a Safety-Net Community Hospital | In hospital ; Outpatient | Bundle Car Payment |
| Li & AL-Amin 2021 | The interaction between high-level electronic medical record adoption and hospitalist staffing levels: A focus on value-based purchasing | In hospital |  |
| Lee et al. 2023 | Transition to Value-based Healthcare: Development, Implementation, and Results of an Optimal Surgical Care Framework at a National Cancer Instituteâ€“designated Comprehensive Cancer Center | In hospital | Other |
| Lawrence et al. 2020 | Variability in skilled nursing facility screening and admission processes: Implications for value-based purchasing | Outpatient | Value Based Purchasing |
| Lassen et al. 2020 | Do Bundled Payment Programs in Joint Replacement Care Hold Promise for Improving Patient Outcomes? | In hospital |  |
| LaFave et al. 2021 | The Value of Home-Based Primary Care:  Qualitative Exploration of Homebound  Participant Perspective | Outpatient |  |
| Labovitz et al. 2017 | Web-Based Patient Experience Surveys to Enhance Response Rates | In hospital | Other |
| Kumar et al. 2022 | Impact of Hospital-Based Rehabilitation Services on Discharge to the Community by Value-Based Payment Programs After Joint Replacement Surgery | Outpatient | Fee for Service ; Bundle Car Payment |
| Kukreja et al. 2021 | Utilizing time-driven activity-based costing to determine open radical cystectomy and ileal conduit surgical episode cost drivers | In hospital | Time-Driven Activity-Based Costing |
| Koy et al. 2023 | The Flipped Break-Even: Re-Balancing Demand- and Supply-Side Financing of Health Centers in Cambodia |  |  |
| Koster et al. 2023 | Dealing with Time Estimates in Hospital Cost Accounting: Integrating Fuzzy Logic into Timeâ€‘Driven Activityâ€‘Based Costing | In hospital | Time-Driven Activity-Based Costing; Other |
| Koressel et al. 2022 | Profound Impact of Insurance Payor and Socioeconomic Status in Total Hip Arthroplasty Outcomes: Results From a High Volume Tertiary Care Center | In hospital | Bundle Car Payment |
| Koolmees et al. 2022 | Time-Driven Activity-Based Costing Accurately Determines Bundle Cost for Rotator Cuff Repair | Outpatient | Time-Driven Activity-Based Costing |
| King et al. 2021 | Prioritization framework for improving the value of care for very low birth weight and very preterm infants | In hospital | Other |
| Karim et al. 2021 | Financial Performance of Hospitals in the Appalachian Region Under the Hospital Readmissions Reduction Program and Hospital Value-Based Purchasing Program | In hospital | Pay for Performance; Value Based Purchasing |
| Jones et al. 2019 | Observations on the Medicare Value- Based Ranking of Hospitals During  Fiscal Years 2015 and 2016 | In hospital | Value Based Purchasing |
| Jayakumar et al. 2023 | A Model for Evaluating Total Costs of Care and Cost Savings of Specialty Condition-Based Care for Hip and Knee Osteoarthritis in an Integrated Practice Uni | In hospital ; Outpatient | Time-Driven Activity-Based Costing |
| Jain et al. 2019 | Strategies for Delivering Value-Based Care:  Do Care Management Practices Improve  Hospital Performance? | In hospital | Value Based Purchasing |
| Ingraham et al. 2016 | Reductions in High-End Imaging Utilization With Radiology Review and Consultation | Outpatient |  |
| Hoong et al. 2023 | Impact of the value driven outcomes  program among cataract surgery patients  in Singapore: an interrupted time series analysis | In hospital |  |
| Hennick et al. 2013 | Value-based healthcare in Lynch syndrome | In hospital |  |
| Harris et al. 2019 | Examining and Understanding Value: The Cost of Preoperative Characteristics, Intraoperative Variables and Postoperative Complications of Minimally Invasive Partial Nephrectomy | In hospital |  |
| Harold et al. 2019 | Single-Use Custom Instrumentation in Total  Knee Arthroplasty: Effect on In-Hospital  Complications, Length of Stay, and Discharge  Disposition | In hospital |  |
| Hale et al. 2021 | Improving Medication Adherence in an ACO Primary Care Office With a PharmacistLed Clinic: A Report From the ACORN SEED | Outpatient | Value Based Purchasing |
| Gronbeck & Feng 2023 | Performance and Quality Measure Selection by Mohs Surgeons in the 2020 Merit-Based Incentive Payment System | In hospital | Pay for Performance |
| Grabowski et al. | The Impact of Nursing Home Pay-for- Performance on Quality and Medicare Spending: Results from the Nursing Home Value-Based Purchasing Demonstration | Other | Pay for Performance |
| Goretti et al. 2020 | Value-Based Healthcare and Enhanced Recovery After Surgery Implementation in a High-Volume Bariatric Center in Italy | In hospital |  |
| Glasgow et al. 2019 | Hospital palliative care consult improves value- based purchasing outcomes in a propensity  scoreâ€“matched cohort | In hospital | Fee for Service ; Value Based Purchasing |
| Ghisleni et al. 2023 | Value-based health care in heart failure: Quality of life and cost analysis | Outpatient | Time-Driven Activity-Based Costing |
| Ganske et al 2021 | Time-Driven, Activity-Based Costing of  Presurgical Infant Orthopedics: A Critical  Component of Establishing Value of Latham  Appliance and Nasoalveolar Molding | In hospital | Time-Driven Activity-Based Costing |
| Galvez et al. 2020 | Value-Based Healthcare in Ostomies | In hospital | Other |
| Gabriel et al. 2019 | Value-based healthcare analysis of joint  replacement surgery for patients with  primary hip osteoarthritis | In hospital | Other |
| Gabe-Walters et al. 2021 | Lymphoedema care in the mobile unit: a service evaluation of patient experience  and attendance rates | mobile care |  |
| Fortmann et al. 2020 | Care Team Integration in Primary Care Improves One-Year Clinical and Financial Outcomes in Diabetes: A Case for Value-Based Care | Outpatient |  |
| Fong et al. 2022 | Medicaid long-term care workforce training intervention and value-based payment metrics | Outpatient | Value Based Purchasing |
| Fidanza et al. 2022 | What Is the Inpatient Cost of Hip Replacement? A Time-Driven Activity Based Costing Pilot Study in an Italian Public Hospital | In hospital | Time-Driven Activity-Based Costing |
| Feizpour et al. 2021 | Enhanced recovery in liver transplantation: A value-based approach to complex surgical care | In hospital |  |
| Farley et al. 2023 | Implementation outcomes associated with a value-based care model of comprehensive medication management in community pharmacies | Outpatient | Fee for Service ; Pay for Performance |
| Etges et al. 2022 | Telemedicine Versus Face-to-Face Care in Ophthalmology: Costs and Utility Measures in a Real-World Setting | Outpatient | Time-Driven Activity-Based Costing |
| Etges et al. 2022 | Moving the Brazilian ischaemic stroke pathway to a  value-based care: introduction of a risk-adjusted cost  estimate model for stroke treatment | In hospital | Time-Driven Activity-Based Costing |
| Ennis et al. 2019 |  | In hospital |  |
| Ellis et al. 2017 | Refining Our Understanding of  Value-Based Insurance Design and  High Cost Sharing on Children | In hospital | Other |
| Dolce et al. 2020 | Interprofessional value-based health care: Nurse practitioner-dentist model | Outpatient |  |
| Dohmen et al. 2022 | Implementing value-based healthcare using a digital health exchange platform to improve pregnancy and childbirth outcomes in urban and rural Kenya | Outpatient | Bundle Car Payment |
| Depla et al. 2022 | Patient-Reported Outcome and Experience Measures in Perinatal Care to Guide Clinical Practice: Prospective Observational Study | In hospital |  |
| Demedts et al. 2021 | Clinical implementation of value based healthcare: Impact on outcomes for  lung cancer patients | In hospital |  |
| Dekkers et al. 2022 | Costs and Its Determinants in Pituitary Tumour Surgery | In hospital |  |
| de Jong et al. 2020 | Cost-effectiveness of Telemedicine-directed Specialized vs Standard Care for Patients With Inflammatory Bowel Diseases in a Randomized Trial | In hospital, tele/videoconference | Fee for Service |
| Daruwalla et al. 2023 | INTRAVITREAL ANTI-VASCULAR ENDOTHELIAL GROWTH FACTOR PHARMACEUTICAL UTILIZATION AND FINANCIAL IMPACT IN A VALUE-BASED CARE PROGRAM | Outpatient | Fee for Service ; Shared Savings Programs |
| Daras et al. | Nearly One In Five Skilled Nursing Facilities Awarded Positive Incentives Under Value-Based Purchasing | Outpatient | Value Based Purchasing |
| Daniels et al. 2022 | Five yearsâ€™ experience with value-based  quality improvement teams: the key factors  to a successful implementation in hospital care | In hospital | Fee for Service |
| daSilvaEtges et al. 2022 | Time-driven activity-based costing as a strategy to  increase efficiency: An analyses of interventional  coronary procedures | In hospital | Time-Driven Activity-Based Costing |
| Cwalina et al. 2022 | Group practice in Urology:  A cross-sectional analysis  over 8years (2014â€“2021) | Outpatient |  |
| Cramer-VanDerWelle et al. 2021 | â€˜Care for Outcomesâ€™: systematic  development of a set of outcome  indicators to improve patient-relevant  outcomes for patients with lung cancer | In hospital |  |
| Cohen et al. 2021 | Surgical Management of Obesity in Brazil: Proposal for a Value-Based Healthcare Model and Preliminary Results | In hospital | Bundle Car Payment |
| Chiu et al. 2022 | Association of the Medicare Value-Based Purchasing Program With Changes in Patient Care Experience at Safety-net vs Nonâ€“Safety-net Hospitals | In hospital | Value Based Purchasing |
| Chen et al. 2017 | Financial Performance of Hospitals in the Mississippi Delta Region Under the Hospital Readmissions Reduction Program and Hospital Value-based Purchasing Program | In hospital | Value Based Purchasing |
| Chatterjee et al. | Patient Experience in Safety-Net Hospitals | In hospital | Value Based Purchasing |
| Chatfield et al. 2019 | Bending the cost curve: time series  analysis of a value transformation  programme at an academic  medical centre | In hospital | Time-Driven Activity-Based Costing |
| Chatfield 2016 | Value-Based Purchasing | In hospital | Value Based Purchasing |
| Calderon et al. | Questionable validity of the catheter-associated urinary tract infection metric used for value-based purchasing | In hospital | Value Based Purchasing |
| Burke et al. 2022 | Skilled Nursing Facility Performance and Readmission Rates Under Value-Based Purchasing | Outpatient | Value Based Purchasing |
| Bueno et al. 2022 | The Clinical outcomes, healthcare resource utilization, and related costs (COHERENT) model. Application in heart failure patients | In hospital | Other |
| Borden & Blustein 2012 | Valuing Improvement in Value-Based Purchasing | In hospital | Pay for Performance |
| Bodar et al. 2020 | Time-Driven activity-based costing identifies opportunities for process efficiency and cost optimization for robot-assisted laparoscopic pyeloplasty | In hospital | Time-Driven Activity-Based Costing |
| Bigger et al. 2021 | Advance Care Planning Protocols and Hospitalization Rates in Home Health Value-Based Purchasing | Outpatient | Fee for Service ; Value Based Purchasing |
| Bensink et al. 2023 | Value-based healthcare in fertility care  using relevant outcome measures for the  full cycle of care leading towards shared  decision-making: a retrospective  cohort study | In hospital |  |
| Basto et al. 2019 | Time-driven activity-based costing to model the utility of parallel induction redesign in high-turnover operating lists | Outpatient | Time-Driven Activity-Based Costing |
| Banerjee et al. 2019 | Association between degree of exposure to the Hospital Value Based Purchasing Program and 30-day mortality: experience from the first four years of Medicareâ€™s pay- for-performance program | In hospital | Pay for Performance |
| Ayoube et al. 2023 | Redefining â€œValueâ€ in Surgery: Development of a Comprehensive Value Score for Outpatient Endocrine Surgery | Outpatient | Fee for Service |
| Artenstein et al. 2021 | An Integrated Mobile Acute Care Service Enhances Value | mobile care | Fee for Service ; Value Based Purchasing |
| Arenchild et al. 2020 | Do We Get What We Pay For?  Examining the Relationship Between  Payments and Clinical Outcomes in  High-Volume Elective Surgery in a  Commercially-Insured Population | In hospital |  |
| Alibrahim Et al. 2021 | Towards value-based healthcare: Establishing  baseline pharmacy care costs for diabetes  management | Outpatient | Time-Driven Activity-Based Costing |
| Alibrahim et al. 2022 | Towards value-based healthcare: Establishing  baseline pharmacy care costs for diabetes  management | Outpatient | Time-Driven Activity-Based Costing |
| Al-Amin et al. 2023 | Were hospitals with sustained high performance more successful at reducing mortality during the pandemicâ€™s second wave? | In hospital | Value Based Purchasing |
| Ahn et al. 2019 | Defining and Optimizing Value in Total Joint Arthroplasty From the Patient, Payer, and Provider Perspectives | In hospital | Bundle Car Payment |
| Ahluwalia 2021 | Improving the efficiency of ankle fracture care through home care and day-surgery units: Delivering safe surgery on a value-based healthcare model | In hospital ; Outpatient ; hospital in the home | Time-Driven Activity-Based Costing |
| Adler-Milstein 2022 | Association of Primary Care Engagement in Value-Based Reform Programs With Health Services Outcomes | Outpatient | Fee for Service ; Value Based Purchasing |
| Abbott 2011 | A Microcosting Approach for Isolated, Unilateral Cleft Lip Care in the First Year of Life | In hospital ; Outpatient | Fee for Service |

**Appendix G:** Barriers and Facilitators of Value Based Healthcare

| Study ID | Title | Barriers | Facilitators |
| --- | --- | --- | --- |
| Sethi et al. 2021 | Utilizing Lean Methodology and Time-Driven Activity-Based Costing Together | TDABC can often only be utilized in very functional systems in which the care pathway of the patient is understood in detail. |  |
| Pestka et al. 2020 | Community pharmacists' perceptions  of acceptability, appropriateness, and  feasibility of a value-based care model for  comprehensive medication management | Difficult to bill due to process barriers and patients that change health insurers frequently |  |
| Panchal et al. 2023 | The implementation of value-based frameworks,  clinical care pathways, and alternative payment  models for cancer care in the United States | Incorporating patient experience, outcomes and care quality into the framework.  Accessing evidence to support clinical pathways of the framework. Incorporating costs in the framework | Producing real- world data for providers, payers and patients |
| Nilsson et al. 2017 | Value-based healthcare as a trigger for improvement initiatives | Demand of lots of IT-related contracts to ensure adequate patient care records.  Difficult to establish outcome measures for treatments other than surgery |  |
| Nguyen et al. 2023 | Is value-based healthcare a strategy to achieve universal health coverage that includes oral health? An Australian case study | Insufficient funding, continuation of traditional fee for service models | Wanting to increase service delivery with limited budget, |
| Maganty et al. 2023 |  | Limited specialty specific measures in MIPS, "topped out" measures hinder the ability of urologists to achieve maximum points for quality. |  |
| Li & AL-Amin 2021 | The interaction between high-level electronic medical record adoption and hospitalist staffing levels: A focus on value-based purchasing | Cost to establish Health Information Technology, delayed maturity of technology | EHR is a facilitator to high performance in a fee for performance model |
| Gronbeck & Feng 2023 | Performance and Quality Measure Selection by Mohs Surgeons in the 2020 Merit-Based Incentive Payment System | Lack of familiarity within the reporting system |  |
| Farley et al. 2023 | Implementation outcomes associated with a value-based care model of comprehensive medication management in community pharmacies | Pharmacies having limited access to Electronic Medical Records |  |
| Dohmen et al. 2022 | Implementing value-based healthcare using a digital health exchange platform to improve pregnancy and childbirth outcomes in urban and rural Kenya | It remains paramount to improve quality of data registries and consistency of data capturing by providers, especially data that is not captured automatically through billing processes such as mortality rates.   the providers are used to being paid on a fee- for-service basis or via capitation, shifting financial risk from payer to provider by introducing full bundled payments was experienced as a bridge too far.  the relatively limited validity of Western patient-reported outcome measures. | Additional training of providers proved key to improve data collection and usage.  Increased transparency of provider performance, benchmarking with other (competing) providers and financial rewarding through bonuses are all factors that impact the willingness of providers to collect and share data |
| de Jong et al. 2020 | Cost-effectiveness of Telemedicine-directed Specialized vs Standard Care for Patients With Inflammatory Bowel Diseases in a Randomized Trial | Broad implementation was hindered due to a lack of evidence |  |
| Daniels et al. 2022 | Five yearsâ€™ experience with value-based  quality improvement teams: the key factors  to a successful implementation in hospital care | Organisation structures struggle to shift to being disease-orientated.  Organisations don't facilitate multidisciplinary care   Integrating VBHC into existing quality improvements is difficult due to having two different approaches at once.  Resistance from health professionals to adapt VBHC concepts  Lack of leadership involvement  Data collection and analysis was considered time consuming and therefore mentioned as a hindering factor | Implementing Patient Reported Outcome Measures as the data collected enriches a VBHC model.  When participants and health professionals outside the improvement teams saw the first results  of VBHC, their believe in the added value of VBHC  increased.  Inspirational medical leadership was an important factor   Seeing differences in outcome data and  discussing ways of working, protocols and procedures  underlying those differences with health professionals  from the other participating hospitals was found inspiring and educational. |
| Borden & Blustein 2012 | Valuing Improvement in Value-Based Purchasing | Barriers to improve are lack of resources, education deficiencies, even with the alternative model a statistically significant effect on locationality proved to be a disadvantage | A more equitable definition on improvement in a VBP model incentivise low performing hospitals to invest in quality improvement and process more than high performers. |
| Ayoube et al. 2023 | Redefining â€œValueâ€ in Surgery: Development of a Comprehensive Value Score for Outpatient Endocrine Surgery | Limited application of the proposed value score equation  The proposed value score equation is only relevant to a certain medical condition and intervention |  |
| Artenstein et al. 2021 | An Integrated Mobile Acute Care Service Enhances Value | Operational efficiency negatively impacted to ensure reduction in unnecessary Emergency, Ambulance or Hospital Admissions. Integrating the primary care with the mobile provider through Electronic Health Records was an ongoings challenge | Patients in Value based models returned the greatest cost saving |
| Adler-Milstein 2022 | Association of Primary Care Engagement in Value-Based Reform Programs With Health Services Outcomes | Voluntary participation in reform programs is low. There is no systematic program synergy due to complex requirements |  |
